# Immunosenescence and Cytomegalovirus: Exploring Their Connection in the Context of Aging, Health, and Disease

**DOI:** 10.3390/ijms25020753

**Published:** 2024-01-06

**Authors:** Ludmila Müller, Svetlana Di Benedetto

**Affiliations:** Max Planck Institute for Human Development, Lentzeallee 94, 14195 Berlin, Germany

**Keywords:** aging, immunosenescence, cytomegalovirus, inflammaging, CMV latency, COVID-19, SARS-CoV-2, long COVID, age-related diseases

## Abstract

Aging induces numerous physiological alterations, with immunosenescence emerging as a pivotal factor. This phenomenon has attracted both researchers and clinicians, prompting profound questions about its implications for health and disease. Among the contributing factors, one intriguing actor in this complex interplay is human cytomegalovirus (CMV), a member of the herpesvirus family. Latent CMV infection exerts a profound influence on the aging immune system, potentially contributing to age-related diseases. This review delves into the intricate relationship between immunosenescence and CMV, revealing how chronic viral infection impacts the aging immune landscape. We explore the mechanisms through which CMV can impact both the composition and functionality of immune cell populations and induce shifts in inflammatory profiles with aging. Moreover, we examine the potential role of CMV in pathologies such as cardiovascular diseases, cancer, neurodegenerative disorders, COVID-19, and Long COVID. This review underlines the importance of understanding the complex interplay between immunosenescence and CMV. It offers insights into the pathophysiology of aging and age-associated diseases, as well as COVID-19 outcomes among the elderly. By unraveling the connections between immunosenescence and CMV, we gain a deeper understanding of aging’s remarkable journey and the profound role that viral infections play in transforming the human immune system.

## 1. Introduction

Aging is an inevitable part of the human experience, a journey marked by the passage of time that brings with it profound changes across various aspects of life. Among the myriad of alterations that accompany the process of growing older, the impact on the immune system stands out as a particularly significant and multifaceted phenomenon [1,2,3]. Immunosenescence, the gradual and complex shift in immune function associated with aging [1,4,5,6,7], has emerged as a critical area of scientific investigation, raising crucial questions about its implications for health, disease, and longevity.

The immune system, a remarkable defense network of tissues, cells, and molecules, plays a crucial role in protecting the body from pathogens, such as bacteria, viruses, and other potential threats. Throughout life, it adapts to new challenges, learns to recognize invaders, and forms immunological memories. However, as individuals navigate the journey of aging, this once-vigilant guardian undergoes a transformation [1,8], rendering the host more vulnerable to infections, impairing its ability to respond effectively to novel threats [6,9], and exacerbating the risk of various age-associated diseases, including cancer, cardiovascular conditions, and neurodegenerative disorders.

A remarkable feature of immunosenescence is its dynamic interplay with cytomegalovirus, a ubiquitous human pathogen [10,11,12]. CMV, a member of the herpesvirus family, has the remarkable capacity to establish lifelong persistence in the human host, often remaining asymptomatic. Although this virus coexists with the host for decades, its presence has been linked to profound changes in the immune landscape. Over the years, researchers have worked to investigate the role of CMV in shaping immune responses and raising questions about the virus’s contributions to both the aging process and age-related diseases [13,14,15,16,17,18]. This review aims to provide an overview of the evidence, shedding light on the multifaceted relationship between CMV and immunosenescence. We will explore the impact of CMV on immune cell populations, the potential mechanisms of viral persistence, and the role of chronic infection in the development of age-associated pathologies.

The COVID-19 pandemic, caused by the novel coronavirus SARS-CoV-2, has presented an unprecedented global health crisis. Within the spectrum of those affected, the elderly have emerged as a particularly vulnerable population, facing increased risks of severe disease and mortality [19,20,21]. However, the vulnerability of older individuals to COVID-19 appears to not solely be attributed to age-related factors. Emerging research has underscored the significance of underlying immune conditions, and among them, CMV infection has gained particular attention [10,15,22,23,24]. The consequences of CMV infection in older adults may be extended beyond its impact on the immune system. Some findings suggest that CMV, with its intricate immunological modulatory effects, may exert a significant influence on the course of COVID-19 in older individuals [10,15,23]. In light of this evolving scientific landscape, our review article intends to uncover the possible relationship between CMV-seropositivity and COVID-19 outcomes in aged populations. It aims to delve into the complex connections between CMV infection, immunosenescence, and the pathophysiology of COVID-19. By placing a primary focus on immunosenescence and CMV, this review intends to elucidate the multifaceted nature of this viral interaction and its implications for the health and well-being of older adults.

In the sections that follow, we will navigate through the hallmarks of immunosenescence, uncover the complexities of CMV, and consider the implications of their interactions. We will consider mechanistic insights, potential pathophysiological mechanisms, and the consequences that collectively contribute to a deeper understanding of the interplay between CMV infection and COVID-19 in the elderly. Together, we aim to gain a deeper understanding of how this persistent viral presence shapes the aging immune system, with far-reaching consequences for health and disease in the elderly.

## 2. Unraveling Immunosenescence: Key Concepts and Hallmarks

**Figure 1:**
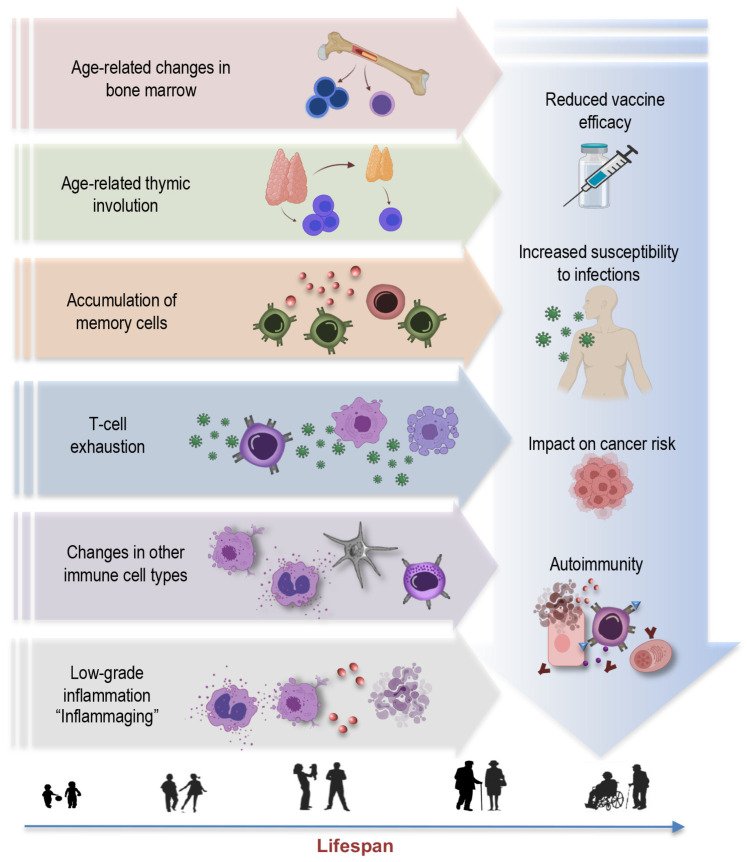
Key concepts and hallmarks of immunosenescence.

Aging is a multifaceted process involving the gradual remodeling of various physiological systems, and the immune system is no exception to these age-related alterations [1,4,19,25,26,27]. This phenomenon, termed immunosenescence, is a prominent feature of aging characterized by the progressive changes in immune function, impacting both innate and adaptive immune responses [28]. The key concepts and hallmarks briefly outlined below represent a selection of central elements (Figure 1) contributing to the complex phenomenon of immunosenescence:

*Age-related changes in bone marrow:* Aging leads to changes in the bone marrow [26,29,30], a vital component of the immune system. These changes include alterations in hematopoiesis, the process of blood cell formation. The bone marrow produces fewer immune precursor cells, impacting the generation of various immune cells, such as lymphocytes and monocytes. This bone marrow shift further contributes to the overall decline in immune function associated with immunosenescence [31]. Understanding these age-related changes in the bone marrow is integral to comprehending the broader concept of immunosenescence.

*Thymus involution:* Another key concept is thymus involution, characterized by the gradual degeneration of the thymus, a primary lymphoid organ central to T-cell development (Figure 1). This process initiates early in life but becomes more pronounced with aging, leading to a significant decline in thymic function [3,7,32,33,34]. This results in a diminished output of new T cells and a skewed T-cell repertoire. The effects of age-related thymic involution are profound, as it directly affects the diversity and functionality of the immune system [32]. The decreased production of naïve T cells compromises the ability to respond effectively to new pathogens and antigens, contributing to an increased susceptibility to infections and a decline in immune surveillance against tumor cells.

*Accumulation of memory cells:* An important hallmark feature of immunosenescence is the accumulation of memory T cells and B cells within the aging immune system [5,7,9,11,29,34,35,36]. These memory cells, formed in response to previous encounters with pathogens, are crucial for mounting rapid and effective immune responses upon re-exposure. However, with advancing age, there is a notable shift in the composition of the immune cell repertoire. The proportion of memory T cells increases, often at the expense of naïve cells, altering the balance within the immune system. Despite an initial increase in memory cells during early life, these cells also undergo senescent changes later in life, with hallmarks such as the loss of CD28 and the accumulation of highly differentiated effector memory T cells [3,37,38]. This skew towards memory cells may compromise the ability to respond to novel pathogens and antigens, impairing the adaptability and robustness of immune defenses in older individuals.

*T-cell exhaustion:* T-cell exhaustion is a key concept within the realm of immunosenescence [39]. This phenomenon refers to a state where T cells lose their functional capabilities, particularly in the context of chronic infections or prolonged exposure to antigens. T-cell exhaustion is characterized by reduced proliferative potential, diminished cytotoxicity, impaired cytokine secretion, and heightened expression of inhibitory receptors, such as programmed cell death protein 1 (PD-1), killer cell lectin-like receptor G1 (KLRG1), and CD57. In the context of aging and chronic viral infections like CMV, the expansion of exhausted T cells, particularly CD28^-^ T cells, is a hallmark of immunosenescence [40]. These cells may retain some cytotoxicity and ability to produce Th1 cytokines, but their overall functional decline contributes to weakened immune responses. Understanding T-cell exhaustion is crucial, as it has implications not only for immunosenescence, but also for diseases associated with chronic infections and aging.

*Changes in other immune cell types:* Immunosenescence extends beyond T cells, affecting various immune cell populations [41]. For instance, natural killer (NK) cells, crucial for the defense against infected or malignant cells, also experience alterations with age. The functional decline of NK cells may result in reduced surveillance against transformed or infected cells [4,6,41,42,43], potentially contributing to higher cancer susceptibility in older individuals. Myeloid cells, including macrophages and dendritic cells, can become more prone to pro-inflammatory activation, leading to the elevated production of pro-inflammatory cytokines such as interleukin-6 (IL-6), tumor necrosis factor-alpha (TNF-α), and interleukin-1 beta (IL-1β). This shift in their functional profile contributes to the low-grade chronic inflammation associated with aging. This pro-inflammatory state also has implications for the functioning of other immune cells, potentially disrupting the immune response to infections and vaccinations [6,41,44,45].

*Inflammaging:* Inflammaging is a central concept in the realm of immunosenescence, representing the chronic, low-grade inflammation that characterizes the aging immune system [25,46]. This persistent inflammatory background, often driven by factors such as cellular stress, senescent cells, and the secretion of proinflammatory molecules, plays a pivotal role in the pathogenesis of various age-associated conditions, including cardiovascular diseases, cancer, and neurodegenerative disorders [7,25,46,47]. Understanding the concept of inflammaging is crucial in elucidating the multifaceted landscape of immunosenescence, as it underscores the intricate interplay between immune aging and age-related diseases, offering potential avenues for therapeutic interventions to mitigate the impact of chronic inflammation on overall health in the elderly.

*Reduced vaccine efficacy:* One of the concerning consequences of immunosenescence is its impact on vaccine responses [48,49]. As the aging immune system becomes less responsive and adaptable, vaccines may be less effective in providing adequate protection [50,51]. This phenomenon is particularly relevant for diseases such as influenza and COVID-19, where vaccines are crucial for preventing severe illness and complications [52,53,54]. Reduced vaccine efficacy in older individuals not only places them at higher risk for vaccine-preventable diseases, but may also hinder efforts to establish herd immunity, as the collective protection of the population depends on a sufficient number of individuals developing immunity through vaccination.

*Increased susceptibility to infections:* Immunosenescence also renders older individuals more vulnerable to a wide range of infections [55]. The diminished immune responses, especially in the adaptive immune system, make it harder for the body to fend off invading pathogens effectively [49]. This heightened susceptibility not only leads to a higher incidence of common infections—like respiratory illnesses—but also increases the risk of developing severe and potentially life-threatening infections. In the context of the COVID-19 pandemic, older adults have been disproportionately affected, experiencing higher rates of severe illness and mortality [56,57,58,59,60]. Additionally, infections, such as pneumonia and urinary tract infections, become more prevalent in older age, often resulting in longer hospital stays and slower recovery times [61,62]. Furthermore, the increased susceptibility to infections can have a cascading effect on overall health, potentially exacerbating age-related comorbidities and contributing to an increased burden of illness in the elderly population.

*Impact on cancer risk:* One consequence of immunosenescence is the diminished ability of the immune system to surveil and eliminate cancerous cells efficiently. This compromised immunosurveillance facilitates the initiation and progression of various cancers in elderly individuals [63,64,65]. Furthermore, chronic viral infections, such as CMV, which becomes more prevalent with age, have been linked to an elevated risk of certain cancers. CMV, in particular, may exacerbate immunosenescence and contribute to the development of malignancies. In addition to promoting tumor growth, the proinflammatory environment induced by immunosenescence can support the proliferation of cancer cells and facilitate their spread. This complex interplay between immunosenescence, chronic infections, and cancer underscores the importance of understanding the role of aging immune system in oncogenesis.

*Autoimmunity:* Paradoxically, while immunity against infections weakens, the risk of autoimmune diseases increases with age [66,67]. This dual impact highlights the complexity of immunosenescence. Autoimmunity refers to a condition in which the immune system mistakenly targets and attacks the body’s healthy cells and tissues [68]. As individuals age, changes in immune regulation mechanisms can lead to a breakdown of self-tolerance, resulting in autoimmune reactions [6,69]. The interplay between immunosenescence and autoimmunity has implications for the development of autoimmune disorders in older populations [6,47,70,71]. Diseases like rheumatoid arthritis, systemic lupus erythematosus, and autoimmune thyroid diseases are more prevalent in elderly individuals, and immunosenescence is believed to contribute to these conditions [72].

In conclusion, immunosenescence is a complex and multifaceted process involving alterations in the function and composition of the immune system that have far-reaching implications for overall health. Revealing these hallmarks is essential for understanding the role of immunosenescence in health and disease, as well as its complex relationship with chronic viral infections like cytomegalovirus. In the upcoming sections, we will explore these relationships more deeply, defining the intricate interplay between immunosenescence and cytomegalovirus.

## 3. Cytomegalovirus: The Silent Companion of Aging

Cytomegalovirus, a member of the Herpesviridae family, is a ubiquitous human pathogen with a remarkable prevalence worldwide. It infects between 40% and 95% of the global population, depending on geographic region and socio-economic factors [10,11,12]. CMV is typically acquired early in life, with primary infection often occurring during childhood or adolescence. Importantly, the majority of CMV infections in immunocompetent individuals remain asymptomatic, resulting in a latent and often lifelong viral presence.

### 3.1. Mechanisms of Latency and Reactivation

After primary CMV infection, the virus enters a latent phase where it persists in specific organ sites, mainly in hematopoietic progenitor cells and cells within the myeloid lineage. During latency, active genome replication and the production of viral progeny are not detectable, but residual transcriptional activity can be identified in several viral gene loci. This phenomenon is sometimes referred to as “sleepless latency.” CMV’s ability to establish latency and reactivate intermittently is a key feature of its persistence in the host [73].

The molecular mechanisms responsible for CMV establishing latency and its reactivation remain areas of active research. Multiple studies have made progress towards understanding how human CMV regulates latency, particularly in CD34^+^ progenitor cells in the bone marrow [74]. It was proposed that latency may be achieved through specific mechanisms of transcriptional silencing that vary depending on the cell type. On the other hand, reactivation can be induced through pathways activated by common triggers such as inflammation, infection, and injury, which are found in multiple cell types. Additionally, the differentiation of myeloid cells into dendritic cells can contribute to reactivation [74]. This highlights the complex relationship between CMV and the host immune response, where the virus exploits cell type-specific gene regulation mechanisms to establish latency and spread infection throughout the body. The influence of the inflammatory environment associated with aging, including the presence of inflammatory cytokines such as IL-6 and TNF-α, might play a role in triggering CMV reactivation in older individuals, occasionally leading to low-level viremia.

Moreover, immunosenescence itself may create an environment that might not only facilitate chronic infections like CMV, but also contribute to the reactivation of latent viruses [74,75]. The bidirectional relationship between immunosenescence and CMV involves complex mechanisms that warrant in-depth exploration. As mentioned before, immunosenescence is closely linked to inflammaging, and CMV infection may intensify this inflammatory environment by promoting the secretion of proinflammatory cytokines. In turn, inflammaging further accelerates immunosenescence, creating a positive feedback loop. This proinflammatory milieu is conducive to CMV reactivation, as the virus thrives in inflammatory conditions.

Immunosenescence is characterized by the progressive decline in T-cell function, including exhaustion, a state where T cells lose their ability to respond effectively to persistent viral infections. CMV, known for establishing lifelong latency, takes advantage of the compromised T-cell responses during immunosenescence. The persistence of CMV-specific T cells, particularly CD8^+^ T cells, contributes to the chronic immune activation seen in aging individuals [75].

Additionally, age-related changes in immune cell populations, such as the expansion of memory T cells, contribute to the permissive environment for CMV reactivation. The altered balance of immune cells—a hallmark of immunosenescence—provides favorable conditions for latent viruses like CMV to undergo reactivation, leading to episodic shedding and potential clinical manifestations.

The bidirectional interaction also involves epigenetic modifications. Immunosenescence-associated changes in DNA methylation and histone modifications may influence the regulation of CMV genes during latency [6]. Conversely, CMV-induced alterations in host cell epigenetics can impact the overall aging process and immune responsiveness.

Furthermore, immunosenescence affects antigen-presentation pathways crucial for recognizing and responding to viral infection [75]. CMV, with its ability to manipulate host cell machinery, may exploit these alterations in order to evade immune surveillance during latency. This interplay between immunosenescence-related changes and CMV strategies underscores the complexity of their bidirectional relationship.

Understanding the bidirectional dynamics between immunosenescence and CMV reactivation is essential for unraveling the complexities of aging-related immune dysfunction. Further research should explore the molecular and cellular intricacies of this relationship, searching for potential therapeutic interventions that target both immunosenescence and CMV in order to promote healthier aging and mitigate age-associated diseases.

It is necessary to emphasize that our understanding of virus–host interactions remains constrained not just by the complex interplay between CMV and the immune system, but also by the absence of an animal model that replicates human CMV physiology, as well as gaps in our knowledge regarding numerous proteins encoded by the CMV genome [76]. Understanding these aspects of CMV, from its complex relationship with the aging immune system to the mechanisms of latency and reactivation, is essential in order to grasp its potential impact on human health, especially in the context of aging and age-related diseases.

### 3.2. Sites of CMV Latency

After the resolution of the primary infection, CMV establishes latency in various organs and tissues throughout the human body [77]. The latent CMV genome, residing in various organs, serves as the molecular foundation for CMV reactivation across multiple organ systems [78,79]. These sites of latency are where the virus remains dormant, without causing active infection or manifest symptoms. The exact sites of CMV latency in humans can be difficult to pinpoint with accuracy. Researchers often turn to primary human tissue and cell culture models, along with the utilization of animal models to study CMV latency. Despite some limitations, these approaches remain a valuable tool for researchers, allowing us to better understand the virus’s behavior and interactions within different anatomical sites [80].

**Figure 2:**
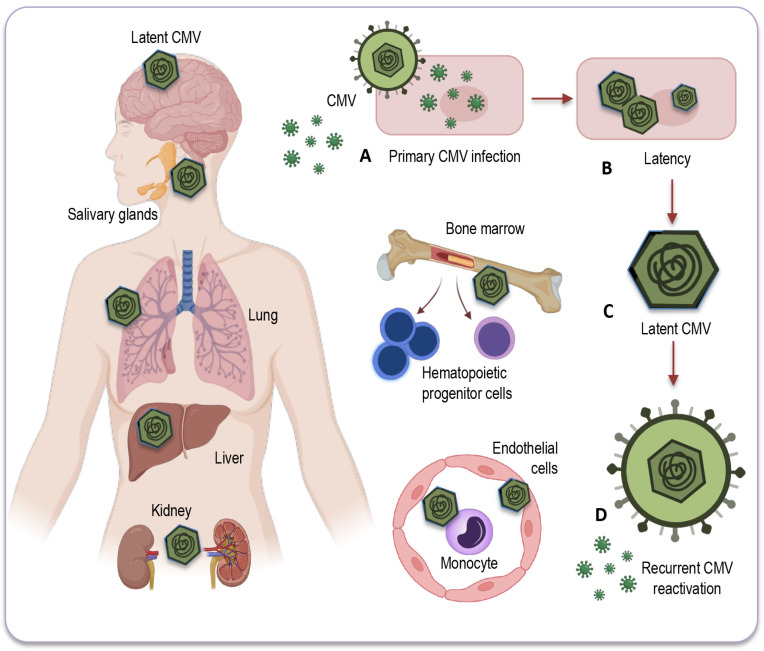
Potential sites of CMV latency. Following the resolution of the primary infection (**A**), CMV enters a latent state (**B**) within various human organs and tissues. This latent CMV genome (**C**) acts as the molecular basis for the reactivation of CMV (**D**) across multiple organ systems. CMV’s primary site of latency is within peripheral blood monocytes and hematopoietic progenitor cells, acting as a viral reservoir for prolonged persistence in the host. Additionally, CMV may be detected in a range of bodily tissues and organs, including the salivary glands, liver, kidneys, brain, and lungs, where reactivation and associated diseases may occur, particularly in individuals with compromised immune systems. CMV: Cytomegalovirus.

CMV primarily establishes latency in peripheral blood monocytes and hematopoietic progenitor cells [81,82]. These cells serve as a reservoir for the virus, allowing it to persist in a latent state within the host (Figure 2).

CMV can also be found in various tissues and organs throughout the body, including the liver [83], kidneys [74], spleen, and lungs [84], where it may reactivate and cause disease under certain conditions, especially in individuals with weakened immune systems. However, while CMV can infect some solid organs and tissues, they are not typically considered a site for CMV latency [78]. The exact nature of CMV’s presence and activity in solid organs remains a topic of ongoing research and discussion.

It is important, therefore, to note that some of the sites depicted in Figure 2, and briefly described below, can be hypothetical in humans, as identifying these locations with certainty can be challenging due to the complexities of CMV latency and analytical difficulties in precisely identifying them, relying on models and animal studies, the diagnostic tools and test methods available so far.

*Myeloid progenitor cells:* CD34^+^ progenitor cells in the bone marrow are considered important sites of CMV latency in humans. Reactivation from these cells can contribute to viral dissemination.

*Monocytes and macrophages:* Monocytes can serve as a reservoir for CMV, harboring the latent virus. CMV may reactivate in these cells and contribute to viral distribution.

*Endothelial cells:* CMV has also been found to establish latency in endothelial cells, which form the lining of blood vessels. This may have implications for vascular health and disease in elderly population.

*Salivary glands:* CMV can persist in the salivary glands, which contributes to its transmission through saliva. Reactivation in the salivary glands can lead to viral shedding and potential transmission of the infection.

*Renal (kidney) tubules:* CMV can establish latency in the renal tubules of the kidneys, which may play a role in the persistence of the virus in the body.

*Liver:* Some research has suggested that the liver could potentially serve as a site for CMV latency, but the details and prevalence of this phenomenon may vary among individuals. Further studies are needed to fully understand the extent and implications of CMV latency in the liver.

*Neural tissue and brain:* CMV’s ability to infect neural tissues, including the brain, has been less investigated. As CMV DNA has also been detected in non-immunocompromised individuals, the virus can undergo reactivation from latency, potentially leading to neurological and neurodegenerative disorders in elderly people.

It is important to note that CMV can possibly reactivate from these sites of latency when the host’s immune system is compromised or weakened. This reactivation can lead to the shedding of the virus and potentially cause symptomatic CMV infections, especially in individuals with weakened immune responses, such as the elderly or immunocompromised individuals. Exploring the sites of CMV latency and its reactivation conditions are vital for developing strategies to manage CMV infections and their potential impact on health, especially in the context of immunosenescence and age-related diseases. Understanding the sites of CMV latency and the mechanisms triggering its reactivation provides a crucial foundation for delving into the upcoming discussion on the intricate interplay between CMV and immunity.

## 4. The Complex Interplay: CMV and Immunity

### 4.1. Age-Related Modulation by CMV

The interaction of CMV with the human immune system is complex, multifaceted, and poorly understood [85]. While primary CMV infection in healthy individuals is usually subclinical or results in mild, flu-like symptoms, the immune response to CMV infection is robust and clear. It elicits a strong, long-lasting, and highly differentiated T-cell response, particularly in older children and adults [86]. CMV-specific memory T cells may become a significant portion of the immune repertoire, and this ongoing immune alteration may impact the overall functionality of the immune system.

In its role of shaping the immune system, CMV presents a unique and complex relationship with the aging immune system. While it is often considered a pathogen due to its potential to cause disease upon primary infection, particularly in infants and immunocompromised individuals, its role in healthy, immunocompetent adults is more nuanced [49]. This dual role is especially evident in CMV-positive young adults, where CMV infection can have both benefits and disadvantages. In particular, CMV infection in young adults typically results in highly differentiated and sensitized T cells specific to the virus, which can rapidly respond to CMV reactivation. This enhanced T-cell population could offer several advantages, including viral control, where CMV-specific T cells can efficiently control CMV reactivation, preventing symptomatic infections. Moreover, CMV-specific T cells may cross-react with other pathogens, providing an immunological benefit when encountering new infections. However, the presence of a highly differentiated T-cell population may also have drawbacks. The continuous activation and differentiation of CMV-specific T cells can lead to immune exhaustion and impaired immune response [87,88].

In the context of aging, the relationship between CMV and the immune system is even more complicated. On the one hand, CMV-specific T cells persist for decades in response to latent CMV infection, maintaining an elevated, specialized, and vigilant population of immune cells. The circulating CMV-specific T cells are often characterized by markers of senescence, such as the loss of CD28 expression, the accumulation of KLRG1 and CD57 markers, and telomere shortening. These hallmarks are typical of immunosenescence and can, in part, be attributed to CMV [89]. On the other hand, the chronic interaction of CMV with the aging immune system raises questions about potential negative consequences. Some evidence suggests that CMV-specific T cells may exhibit features of exhaustion and decline in functionality, which could further contribute to immunosenescence, ultimately affecting the host’s ability to respond effectively to other infections and possibly impacting overall health in aged individuals.

In various studies, it has been widely acknowledged that cytomegalovirus plays a significant role in driving immunosenescence. As mentioned before, one of the prominent features of immunosenescence is the expansion of CD28^-^ T cells, which has been equally recognized as a hallmark of chronic CMV infection. CD28, a co-stimulatory molecule on naïve CD4 and CD8 T cells, is permanently lost during antigen-driven differentiation to a terminal phenotype, indicating replicative senescence [89]. While aging has historically been considered a key factor in this expansion, it is now clear that CMV infection may be a primary driver of this phenomenon. It has been shown that CMV-seropositive individuals exhibit up to a twelve-fold increase in CD4^+^CD28^−^ T cells and a two-fold increase in CD8^+^CD28^−^ T cells compared to CMV-seronegative individuals. Remarkably, this effect is only marginally influenced by age when CMV infection is present [89,90].

The CD8^+^ T-cell response to CMV is notably extensive, targeting a wide range of viral peptides. This response exhibits the unique characteristic of a continuous, long-term expansion of antigen-specific CD8 memory T cells, a phenomenon known as memory inflation. In fact, the CD8^+^ T-cell response to specific epitopes of this virus can constitute up to 20% of the total memory T-cell population, and the cumulative response to all CMV epitopes in humans is estimated to occupy 50% or more of the entire CD8^+^ memory T-cell pool [91]. This substantial and progressive response has been linked to the accumulation of dysfunctional CMV-specific T cells and potentially shorter lifespans in octogenarians, although the precise nature of this association remains unclear.

Intriguing insights into the complexity of the immune response to CMV in the elderly were demonstrated by Hadrup et al. [92]. They observed significant expansions of CD8^+^ T-cell clones specific to dominant CMV peptides in the majority of octogenarians and nonagenarians in a Swedish population. Notably, these expansions tended to become more pronounced with advancing age. Surprisingly, among individuals who lived to very old age, a different trend emerged. Their T-cell responses to CMV were comparatively lower, and some specific T-cell clonotypes responsive to the virus were even lost [92]. These findings suggest the interesting possibility that survival into extreme old age, including centenarians and beyond, may be linked to an immune system that is less preoccupied with persistent infections.

The concept of extreme longevity and its potential correlation with a diminished emphasis of the immune system on persistent infections opens a captivating avenue for exploration. The observation that individuals reaching very old age exhibit lower T-cell responses to CMV, coupled with the loss of specific responsive T-cell clonotypes [92], suggests a unique immune landscape in this demographic. This divergence from the typical pattern seen in aging individuals prompts a critical inquiry: could the reduction in immune responsiveness to persistent infections be a distinctive feature of those who achieve extreme longevity? Exploring this notion may unravel novel insights into the complex dynamics between aging, immune function, and the ability to reach remarkable lifespans. Investigating whether an immune system less preoccupied with persistent infections contributes to the remarkable resilience observed in individuals who defy the conventional boundaries of aging holds promise for opening new insights into the complex dynamics between aging, immune function, and the ability to achieve extraordinary lifespans.

Thus, CMV’s role in shaping immunity, especially in the context of aging, remains multifaceted. On one hand, it can drive immunosenescence, contributing to age-related pathologies, while, on the other, it may enhance immune responses against certain infections, mostly in young individuals. This dual role emphasizes the need for a comprehensive understanding of the interplay between CMV and immunity. The understanding of the immune system’s dynamics in extreme old age may also open new avenues for exploring the interplay between CMV, longevity, and immune function. Moving forward to the next section, we will explore how CMV may contribute to both inflammation and the aging process.

### 4.2. Interplay between CMV and Immunosenescence: Modulatory Effect of Genetics and Lifestyle

In general, immunosenescence exhibits considerable heterogeneity among individuals, and understanding these diverse trajectories is crucial. This requires a comprehensive exploration, recognizing the influence of various factors, including genetics and lifestyle, in modulating the impact of CMV on immunosenescence [28,93]. Factors such as genetic variations among individuals can influence their susceptibility to infections and the efficiency of their immune responses. Some individuals may carry genetic factors that make them more resilient or more vulnerable to CMV. Genetic predispositions may determine aspects of the immune system’s function, such as the effectiveness of specific immune cells in recognizing and combating the virus.

Genetic diversity contributes to variability in immune system function [93,94,95]. Different individuals may have variations in genes related to immune response pathways, cytokine production, and other immune-related functions. These genetic differences can impact how the body responds to CMV infection [94,96]. For example, genes involved in antigen presentation, such as those encoding human leukocyte antigens (HLA), may determine how effectively the immune system presents viral antigens to T cells. Genetic diversity in these genes can affect the recognition of CMV by the immune system. The genes encoding immune receptors, such as those on T cells, may play a crucial role in recognizing and responding to CMV. Genetic variations in these receptor genes can affect the specificity and strength of the immune response. It is known that interferons are key players in antiviral defense. Genetic variations can impact the efficiency of interferon responses to CMV, affecting the ability to control viral replication. Furthermore, individuals’ variations in genes related to inflammatory pathways may influence the degree of inflammation triggered by CMV. Excessive inflammation can further contribute to immunosenescence and age-related diseases.

Additionally, lifestyle factors, including diet, exercise, and overall health practices, further contribute to this complexity [97,98]. They can modulate the effects of CMV on the immune system and overall health. Epigenetic modifications, which can be influenced by lifestyle factors, play a crucial role in regulating gene expression. Lifestyle choices such as diet, stress, and environmental exposures can modify epigenetic marks, influencing how genes related to immune function respond to CMV and other infections. Lifestyle factors such as unhealthy diet, lack of physical activity, and exposure to environmental toxins can contribute to chronic inflammation [98]. Genetic predispositions to inflammatory responses may further exacerbate the impact of CMV and contribute to the development of age-related diseases.

Thus, unraveling the interplay between CMV, genetics, and lifestyle is essential for a more nuanced comprehension of immunosenescence trajectories, shedding light on personalized approaches to mitigate its effects and promote healthy aging. The interplay between genetics and lifestyle further underscores the complexity of the host–virus interaction, and highlights the importance of adopting a holistic approach to health. From another perspective, this understanding may contribute to the development of tailored approaches for addressing the impact of CMV on the aging immune system.

In the following section, we will provide a concise introduction to the intricate mechanisms by which CMV may actively contribute to the exacerbation of inflammation and accelerate the aging process.

## 5. Role of CMV in Fueling Inflammation and Aging

Beyond its impact on immunosenescence, CMV has increasingly gained attention for its complex role in the shaping of proinflammatory conditions within the host [99], contributing to the development of inflammaging, a state characterized by chronic low-grade inflammation that accompanies aging [8,46]. What mechanisms may facilitate CMV in inducing such a proinflammatory environment?

CMV infection has been linked to the activation of specific signaling pathways associated with inflammation [99,100]. Notably, CMV can stimulate the nuclear factor-kappa B (NF-κB) pathway, a key regulator of proinflammatory gene expression. Activation of NF-κB leads to the production of inflammatory mediators [91] and can activate innate immune responses by triggering pattern recognition receptors (PRRs), such as Toll-like receptors (TLRs). This recognition leads to the production of proinflammatory cytokines, like IL-6 and TNF-α, which are known key players in inflammation. CMV infection can alter immune cell profiles and functions [101]. For instance, as repeatedly discussed before, CMV is associated with an expansion of CD28^−^ T cells, which are functionally less effective but produce more proinflammatory cytokines, fueling inflammation.

Furthermore, CMV can induce senescent cells to produce SASP (senescence-associated secretory phenotype) factors, which include proinflammatory cytokines and chemokines. These factors contribute to a chronic state of inflammation that characterizes inflammaging. As discussed earlier, CMV can also exacerbate immunosenescence, contributing to chronic inflammation. This weakened immune function, coupled with the presence of CMV, may lead to persistent inflammation [102,103]. Additionally, CMV-specific T cells, while still highly cytotoxic, can produce proinflammatory cytokines. This can further contribute to persistent inflammation, especially in older individuals [104].

Taken together, CMV-induced immune alterations can similarly lead to a proinflammatory milieu, with increased production of inflammatory cytokines like IL-1β, IL-6, IL-8, and TNF-α. These cytokines are known drivers of inflammation and can lead to the activation of various immune and non-immune cells, further perpetuating a proinflammatory state. This can potentially initiate a detrimental cycle, resulting in further T-cell exhaustion and dysfunction, thus exacerbating the immune system’s limitations in effectively countering other infections and boosting the progression of immunosenescence. These conditions may collectively create a proinflammatory milieu in CMV-infected individuals, which has implications for age-related diseases, including cardiovascular diseases and cancer, and may even play a role in responses to acute infections like SARS-CoV-2. Therefore, the relationship between CMV and inflammation is a multifaceted and dynamic one, with implications for both immune function and age-related pathologies. Understanding these mechanisms is crucial for unraveling the complex interplay between CMV and inflammation in the context of aging and age-associated conditions. Moving forward to the following section, we will explore the impact of CMV on age-related disorders, shedding light on the multifaceted interplay and the intricate connections between chronic viral infections and the development of various health conditions.

## 6. CMV and Age-Related Diseases

A growing body of evidence suggests that CMV—with its ability to promote immunosenescence—is associated with an increased risk of developing several age-related diseases [105]. The sustained presence of inflammation driven by CMV may contribute to the development and progression of age-related disturbances [102]. Chronic inflammation is a hallmark of conditions like atherosclerosis, neurodegenerative disorders, and cancer. In the context of CMV, immunosenescence and inflammaging could exacerbate the pathogenesis of these diseases, making CMV infection a potential risk factor in aging populations.

### 6.1. CMV and Cardiovascular Diseases

CMV has been recognized as a potential contributor to the development and progression of cardiovascular diseases, a major health concern among the elderly [6,106,107]. This viral infection, often asymptomatic in immunocompetent individuals, can nevertheless have far-reaching consequences on the cardiovascular system. While the mechanisms are not yet fully elucidated, several lines of evidence suggest a significant association between CMV and cardiovascular diseases, particularly atherosclerosis.

One of the key pathways through which CMV may impact cardiovascular health is chronic inflammation [107]. As discussed earlier, CMV can induce a proinflammatory environment, promoting the production of cytokines, particularly IL-6 and TNF-α. These inflammatory molecules play a pivotal role in atherosclerotic plaque formation and progression [108]. They can stimulate endothelial cell dysfunction, attract monocytes and NK cells to the arterial wall, and initiate the transformation of monocytes into foam cells, which are integral to the development of atherosclerotic lesions [109,110]. In these conditions, the persistent activation of CD4^+^CD28^−^ T cells contributes to heightened inflammation, vascular damage, and an increased risk of cardiovascular events, establishing a crucial link between chronic inflammatory states, cytomegalovirus infection, and cardiovascular mortality [111].

Additionally, CMV may contribute to endothelial dysfunction, another hallmark of atherosclerosis [112]. The virus can infect endothelial cells and impair their ability to regulate vascular tone and inflammation [113]. This dysfunction can lead to reduced nitric oxide production, an important vasodilator, and increased production of endothelin-1, a potent vasoconstrictor [114,115]. Furthermore, CMV may promote oxidative stress, an essential player in cardiovascular pathogenesis. The virus can induce the expression of prooxidative enzymes, generating reactive oxygen species (ROS) that damage endothelial cells and lipoproteins. Oxidative stress contributes to the initiation and progression of atherosclerotic lesions [116].

Thus, while the direct impact of CMV on cardiovascular diseases remains an area of ongoing research and debate, the available evidence underscores the potential role of CMV in shaping the proinflammatory, proatherogenic milieu that characterizes many cardiovascular diseases. Understanding these mechanisms is crucial for advancing strategies to mitigate the cardiovascular risk associated with CMV infection, especially among the elderly population.

### 6.2. CMV and Cancer: A Complex Relationship

The relationship between CMV and cancer is complex and multifaceted. CMV has been found in various types of tumors, including breast, ovarian, colon, prostate, sarcomas, neuroblastoma, glioblastoma, and medulloblastoma [117,118,119,120]. However, it is essential to note that the role of CMV in carcinogenesis is far from straightforward.

One aspect of CMV’s interaction with cancer involves its potential oncomodulatory ability [121,122,123,124]. Oncomodulation refers to the process by which a virus influences or promotes carcinogenesis within a host. In the context of CMV, this involves viral gene products with the potential to activate pro-oncogenic pathways [125,126]. Some strains of CMV may possess oncomodulatory roles, and, in specific cellular contexts, can directly promote tumor growth and progression. Correspondingly, the presence of CMV in tumors has raised questions about its role in cancer development [121,127,128]. Still, it remains unclear whether CMV is a causal agent in oncogenesis or whether it infects tumors opportunistically. Furthermore, its presence within tumor tissues may vary widely, with some tumors showing higher CMV DNA levels than others.

In the context of immune control, the impact of CMV on the immune system may favor cancer development. The virus may lead to the accumulation of functionally exhausted T cells, reducing their capacity to eliminate tumor cells. Additionally, CMV-induced proinflammatory environments and inflammaging may contribute to a tumor-promoting milieu. One example of this complex interplay was observed in patients with glioblastoma multiformes who were CMV-positive [129,130]. In such cases, signs of immunosenescence in CD4^+^ T cells have been associated with a poor prognosis, possibly reflecting the influence of CMV [131].

Thus, while the exact role of CMV in cancer remains a subject of ongoing debate, the evidence suggests that its presence within tumor tissues can impact the tumor microenvironment and immune responses. This complex relationship warrants further investigation, as understanding how CMV influences cancer may provide new insights into cancer prevention, diagnosis, and treatment strategies, particularly in the context of aging and immunosenescence.

### 6.3. CMV and Its Potential Role in Neurogenerative Disorders

Neurodegenerative disorders, a group of debilitating conditions affecting the nervous system, are often linked with the process of aging and remain a subject of growing interest. While the precise mechanisms underlying the development of neurodegenerative disorders remain multifaceted, emerging evidence suggests a potential link between CMV and these conditions. Though CMV primarily infects epithelial cells and leukocytes, it can also establish latent infections in various tissues, including neural tissue. CMV DNA has been detected in the brains of some immunocompetent individuals, exhibiting diverse neuropathological changes, often related to cerebrovascular issues [132]. Post-mortem studies of individuals with various neurologic conditions have detected CMV DNA and proteins within brain tissues [132,133], suggesting that CMV can indeed infiltrate the central nervous system (CNS). These findings indicate that, under specific conditions, CMV-induced neuropathology may also manifest in individuals who are not traditionally categorized as immunocompromised.

Infection of the CNS by CMV can lead to long-term viral persistence, which, in turn, may contribute to neuroinflammatory responses and neuronal damage. It has been shown that CMV infection may contribute to cognitive decline [12,134,135,136], elevate the risk of various neurological and psychiatric disorders, including stroke, anxiety, schizophrenia, and bipolar disorder [136,137,138], and has even been suggested as a potential factor in the progression of Alzheimer’s disease [139,140]. Additionally, there is a notable susceptibility among individuals diagnosed with major depressive disorder to the reactivation of CMV. This susceptibility is attributed to the well-established connection between depression, elevated stress levels, and compromised viral immunity, as supported by several investigations [141,142,143].

Moreover, an age-related inflammatory state may trigger the reactivation of latent CMV infection, exacerbating neuroinflammation and potentially impairing cognitive function [10,12,135]. Multiple studies have confirmed the association between CMV infection and cognitive decline in the general population [17,144,145,146,147]. As a neurotropic virus, CMV can induce CNS inflammation by entering brain either through a compromised blood–brain barrier or via peripheral nerve transmission [148]. Significantly, CMV has been found to localize in various brain regions, including the brainstem, diencephalon, and basal ganglia [149], suggesting that CMV could be responsible for CNS damage. Furthermore, in relation to depressive disorders, CMV has been associated with reduced resting-state connectivity, compromised white matter integrity, and altered gray matter volumes [132,150,151], indicating that CMV may induce structural and functional changes in the brains of elderly individuals with depression [152].

Inflammation in general plays a pivotal role in the pathogenesis of many neurodegenerative disorders, including Alzheimer’s disease and Parkinson’s disease [143]. As CMV infection has the potential to induce chronic inflammation within the host, this inflammatory state could act as a catalyst for neurodegenerative processes [153]. As mentioned earlier, CMV can undergo reactivation from latency under certain conditions and this reactivation may involve viral shedding in the CNS. The immune responses generated to viral products during reactivation can further contribute to neuroinflammation, potentially leading to neuronal damage [132,153]. This aspect may be particularly relevant in neurodegenerative disorders, where chronic low-level inflammation is believed to be a contributing factor. It is also conceivable that CMV infection could initiate an autoimmune response, possibly through mechanisms such as molecular mimicry, ultimately resulting in tissue damage [154].

Therefore, it is important to note that the relationship between CMV and neurological disorders, particularly neurodegenerative conditions, is still an area of ongoing research and debate. While the evidence suggests the ability of CMV to establish infections in the CNS and its potential implications in neuroinflammatory processes, further studies are needed to clarify the precise mechanisms and the extent of CMV’s involvement in neurological conditions. Therefore, this complex relationship continues to be a subject of active investigation.

## 7. Impact of CMV on Immune Response to SARS-CoV-2

The influence of CMV extends beyond its specific immune response. Recent research has explored the effects of CMV on immune responses to other pathogens, with a particular focus on SARS-CoV-2, the virus responsible for COVID-19. SARS-CoV-2 is known for its complex interactions with the immune system, and understanding how CMV might influence the immune response to this virus is of great interest.

The correlation between CMV serostatus and the clinical outcomes of COVID-19 suggests that CMV-induced remodeling of the immune system may be involved in the pathogenesis of the SARS-CoV-2 infection [155]. Studies have shown that CMV-positive individuals, while not necessarily more susceptible to SARS-CoV-2 infection, exhibit unique immune responses upon exposure to the virus. Specifically, the presence of CMV-specific CD4 and CD8 T cells is associated with elevated levels of SARS-CoV-2-specific IL-17-producing CD4 and CD8 T cells. This suggests that CMV may skew the immune response to SARS-CoV-2, potentially promoting a Th17-type of response [156]. This immune phenotype can influence inflammation and the course of disease, indicating that CMV-amplified responses to SARS-CoV-2 might play a role in the long-lasting damage associated with COVID-19.

**Figure 3:**
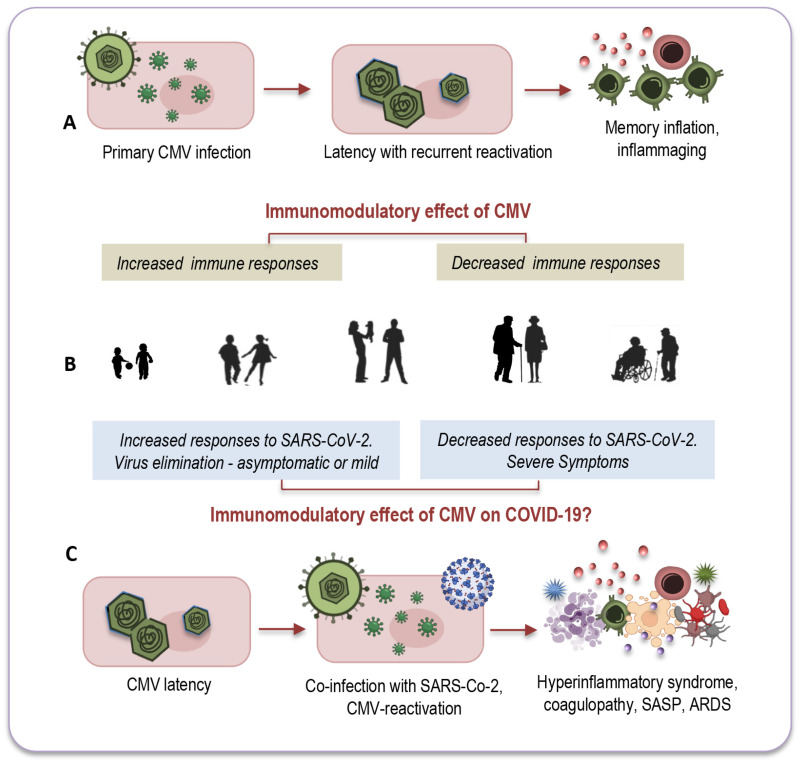
The age-related modulatory effect of CMV. In younger individuals, CMV might increase immune responses ((**A**,**B**) **left**), while in older individuals, it may reduce immune functions ((**A**,**B**) **right**). This dual immunomodulatory effect could also apply to co-infections with SARS-CoV-2 (**C**). Among CMV-seropositive young adults ((**B**) **left**), its presence could stimulate an effective immune response against the new pathogen, efficiently activating both innate and adaptive immune responses. Conversely, co-infection of CMV with SARS-CoV-2 may have a more detrimental impact on elderly individuals ((**B**) **right**, (**C**)) due to immunosenescence and inflammaging, partly attributed to CMV’s lifelong persistence. CMV: cytomegalovirus; SARS-CoV-2: severe acute respiratory syndrome coronavirus 2; COVID-19: coronavirus disease 2019; SASP: senescence-associated secretory phenotype; ARDS: acute respiratory distress syndrome. Modified from: [10].

Another intriguing question that remains unexplored relates to CMV-positive young adults, for whom co-infection with both viruses seems to have less pronounced consequences. There is a growing consensus that the effects of CMV may vary depending on the age of its host. In younger individuals, CMV infection could potentially enhance immune response [10,156], whereas in older individuals it might have a more detrimental impact on immune functions [157], as depicted in Figure 3A,B.

Remarkable insights emerged from a study conducted by Furman and colleagues, shedding light on these age-dependent effects [158]. The study revealed that CMV-seropositive young individuals exhibited improved antibody responses to influenza vaccinations, heightened CD8 T-cell responses, and elevated levels of circulating IFN-γ compared to their CMV-seronegative counterparts. Notably, this research also observed the diminished responses to vaccination commonly seen in elderly individuals, regardless of their CMV serostatus (Figure 3A,B). In a murine model, CMV-seropositive young mice displayed enhanced protection against influenza compared to CMV-seronegative mice, indicating that this dual age-related effect may be a universal phenomenon across vertebrates [10].

It is possible that CMV could exert a similar dual immunomodulatory effect in the context of co-infection with SARS-CoV-2, as illustrated in Figure 3B,C. In CMV-seropositive young adults, the presence of CMV might stimulate an effective immune response against the new pathogen by promptly and effectively activating both innate and adaptive immune responses [10]. This coordinated response could efficiently eliminate the new invader and establish robust immune memory. Conversely, co-infection of CMV with SARS-CoV-2 may have a more profound impact on elderly individuals due to immunosenescence and inflammaging, which, in part, result from the lifelong persistence of CMV (Figure 3B,C).

**Figure 4:**
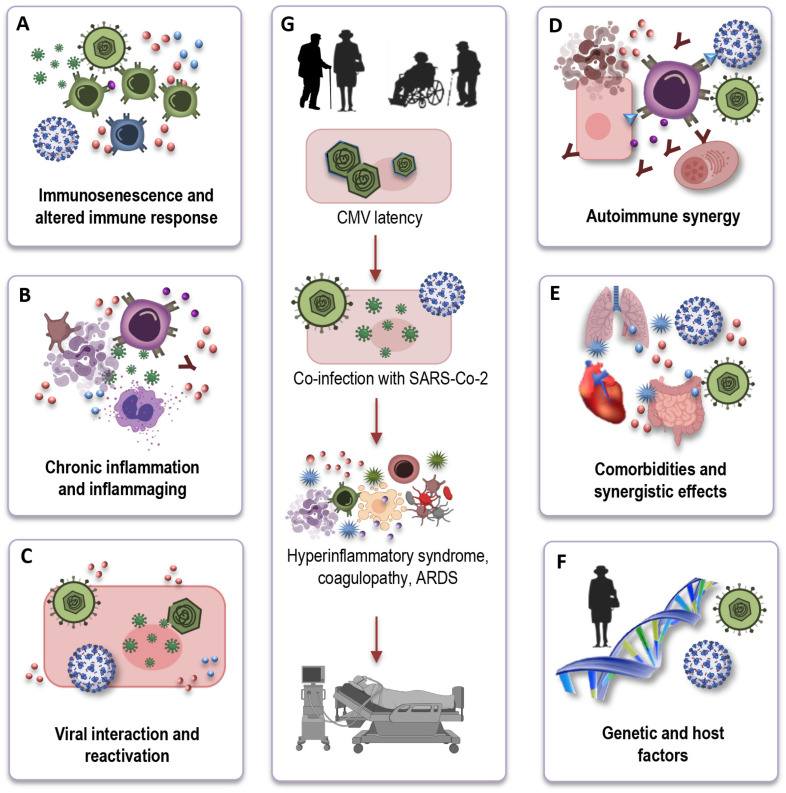
The potential influence of CMV on immune responses to SARS-CoV-2. CMV may hinder the effective immune response to SARS-CoV-2 in older individuals due to immunosenescence (**A**). CMV-related chronic inflammation may exacerbate the cytokine storm in severe COVID-19 cases (**B**). Viral interactions could result in CMV reactivation during COVID-19, complicating immune responses (**C**). CMV-induced autoimmunity and potential SARS-CoV-2-triggered autoimmunity may jointly affect COVID-19 outcomes in the elderly (**D**). CMV may intensify the risk of severe COVID-19 in elderly individuals with comorbidities (**E**). Variability in immune responses, genetics, and CMV strains contributes to heterogeneous COVID-19 outcomes (**F**). Due to the co-infection of SARS-CoV-2 and CMV, older individuals may encounter a hyperinflammatory syndrome, coagulopathy, and ultimately, a deteriorated clinical outcome (**G**). CMV: cytomegalovirus; SARS-CoV-2: severe acute respiratory syndrome coronavirus 2; ARDS: acute respiratory distress syndrome.

Understanding the mechanistic underpinnings of the interaction between CMV-seropositivity and COVID-19 outcomes in the elderly is crucial for unraveling the complexities of this relationship. While the precise mechanisms remain an active area of investigation, several potential pathways and factors can be considered (Figure 4A–F) based on immunological and inflammatory processes:

*Immunosenescence and altered immune response:* CMV infection, particularly in older individuals, exacerbates immunosenescence (Figure 4A). One key mechanism through which CMV may influence COVID-19 outcomes is by altering the immune response. CMV-driven clonal expansion of late-differentiated CD8 T cells may hinder the immune response to SARS-CoV-2 in older COVID-19 patients, potentially causing more severe disease (Figure 4G).

*Chronic inflammation and inflammaging:* CMV infection is associated with chronic low-grade inflammation (Figure 4B). In the context of COVID-19, CMV-induced inflammaging may contribute to the exaggerated cytokine storm observed in severe cases, leading to increased tissue damage and disease severity (Figure 4G).

*Viral interactions and reactivation:* Another intriguing aspect of the interface is the potential for viral interactions (Figure 4C). The inflammatory stress and immune responses triggered by COVID-19 infection could potentially reactivate latent CMV, causing further immunological complications. This bidirectional effect warrants investigation to determine how the two viruses may influence each other’s pathogenesis.

*Autoimmune synergy:* In older individuals, CMV, combined with immunosenescence, can potentially stimulate autoimmune reactions, leading to an overactive immune response. Simultaneously, SARS-CoV-2, the virus responsible for COVID-19, can also induce autoimmunity through mechanisms like molecular mimicry (Figure 4D). The CMV-induced autoimmunity, combined with the potential for SARS-CoV-2 to trigger autoimmunity, could have a synergistic and potentially devastating effect on the outcome of COVID-19 in the elderly.

*Comorbidities and synergistic effects:* Elderly individuals who are CMV-seropositive often have a higher burden of comorbidities, such as cardiovascular disease, diabetes, and hypertension. These comorbid conditions are also known risk factors for severe COVID-19 outcomes. CMV infection may further exacerbate the risk in these individuals, creating a synergistic effect that elevates their susceptibility to severe COVID-19 (Figure 4E).

*Genetic and host factors:* It is important to recognize that individual variability in immune responses, genetic factors, and the specific strain of CMV may all contribute to the heterogeneity in COVID-19 outcomes among CMV-seropositive elderly individuals (Figure 4F). Research efforts are of great importance to elucidate the genetic and host factors that play a role in this complex interaction.

In conclusion, the relationship of CMV with the immune system is multifaceted, with both benefits and disadvantages to CMV-specific immune responses, varying with the age of individuals. While the precise mechanisms linking CMV-seropositivity and COVID-19 outcomes in the elderly continue to be investigated, it is clear that CMV has the potential to influence the immune response, exacerbate inflammation, and interact with other risk factors in older individuals. These complex interactions underline the need for a multifaceted approach to managing and preventing severe COVID-19 in the aging population, particularly those who are CMV-seropositive. Further research is essential to unravel the intricacies of this interaction and guide the development of tailored interventions.

## 8. How CMV May Impact Long COVID?

The impact of CMV on Long COVID, the condition characterized by persistent or lingering symptoms following acute COVID-19 infection, remains unclear. Still, CMV may represent another potential viral candidate that could play a role in the severity of COVID-19 and the development of Long COVID [10,159]. The association of CMV serostatus with COVID-19 clinical outcome suggests a potential role for CMV-induced immune system remodeling in the pathogenesis of SARS-CoV-2 infection [155]. CMV reactivation could contribute to the persistent inflammation often observed in Long COVID patients, who continue to experience symptoms even after SARS-CoV-2 is no longer detectable [159,160,161]. Consequently, it is conceivable that CMV infection may contribute to the pathology of Long COVID by promoting immunosenescence, sparking neuroinflammation, and triggering persistent neuropathological symptoms, particularly in older populations [71,159].

Although the precise interactions between CMV and Long COVID remain elusive, we can anticipate several potential mechanisms, some of which have also already been considered to play conceivable role in the acute phase of infection:

*Immune dysregulation:* In Long COVID, some individuals experience persistent immune activation and inflammatory symptoms. This ongoing immune response may influence the overall immune milieu and exacerbate the immune dysregulation observed in Long COVID. CMV-induced immune alterations could potentially play a role in this phenomenon. In general, Long COVID is characterized by a sustained and dysregulated immune response that extends beyond the acute phase of SARS-CoV-2 infection [71,162,163]. CMV, as a persistent herpesvirus, can further complicate this scenario [10,71,164]. One proposed mechanism involves the potential for CMV to intensify the inflammatory response initiated by SARS-CoV-2, leading to a prolonged and heightened state of inflammation. This sustained inflammation may, in turn, contribute to the persistent symptoms observed in Long COVID [71,165,166]. Additionally, CMV-induced immune senescence and exhaustion could impair the adaptive immune response, further hindering the resolution of SARS-CoV-2 infection. Understanding these nuanced mechanisms is crucial for unraveling the intricate connections between CMV and the lingering immunological consequences of COVID-19.

*Viral interactions:* As mentioned earlier, there is a hypothetical possibility that CMV and SARS-CoV-2 could interact within the host. One aspect of this mechanism involves the potential for one virus to modulate the replication and activity of another [10,71,164]. In the case of CMV and SARS-CoV-2, their coexistence could lead to reciprocal effects. For instance, the inflammatory response triggered by SARS-CoV-2 infection might create conditions conducive to CMV reactivation. Conversely, CMV’s immunomodulatory properties could impact the host’s ability to mount an effective response against SARS-CoV-2. This bidirectional influence highlights the intricate and still incompletely understood nature of viral interactions, shedding light on the complexity of how multiple viruses can shape the host’s immune landscape and influence disease outcomes. However, the specific mechanisms and consequences of such interactions remain largely speculative and require further study.

*Impact on symptom severity:* CMV-induced immunosenescence and chronic inflammation might exacerbate some Long COVID symptoms, especially those related to immune responses and inflammation. For example, fatigue, brain fog, and musculoskeletal symptoms associated with Long COVID could be influenced by the inflammatory environment fostered by CMV [10,71,159,164,167]. Understanding the interplay between CMV-induced immunological changes and the persistence of Long COVID symptoms provides valuable insights into the complex mechanisms underlying the prolonged effects of SARS-CoV-2 infection.

*CMV-induced autoimmunity:* CMV, through its ability to induce persistent inflammation and immune dysregulation, may contribute to the development of autoimmunity. Therefore, one hypothetical mechanism could be CMV- stimulated autoimmunity worsening Long COVID symptoms. The virus can potentially trigger an autoimmune response, possibly via molecular mimicry or other mechanisms. In the context of Long COVID, CMV-induced autoimmunity could play a role in exacerbating the chronic symptoms experienced by individuals recovering from SARS-CoV-2 infection. The persistent immune activation and dysregulation caused by CMV may contribute to the prolonged inflammation and immune-related symptoms characteristic of Long COVID [71]. Thus, in Long COVID, this sustained autoimmune response, combined with efforts to clear the virus, may exacerbate symptoms and contribute to their chronicity. This can lead to a vicious cycle of immune dysregulation, inflammation, and tissue damage, intensifying Long COVID symptoms. Further research is required to confirm these interactions. Understanding these mechanisms is crucial for developing targeted interventions to alleviate the long-term effects of viral infections.

*Prevalence and risk factors:* As discussed earlier, CMV-seropositivity is more common in older individuals. Long COVID has been reported across age groups, but certain risk factors, such as age, can influence its severity. Therefore, age remains a significant risk factor influencing the severity and duration of Long COVID symptoms [164]. In older individuals, where CMV prevalence is higher, the coexistence of CMV may interact with age-related immune changes, potentially leading to more prolonged or severe symptoms. Moreover, the immunomodulatory effects of CMV may exacerbate the inflammatory environment associated with Long COVID, influencing the persistence and intensity of symptoms [159]. Further research is warranted to unravel the intricate interplay between CMV, age, and Long COVID risk factors, shedding light on potential mechanisms that contribute to the varied clinical manifestations observed in individuals experiencing prolonged effects post-SARS-CoV-2 infection.

*CMV reactivation and differential diagnosis:* CMV reactivation or chronic CMV-related symptoms could mimic or imitate some Long COVID symptoms, leading to challenges in accurate diagnosis. Both conditions can manifest with persistent fatigue, cognitive dysfunction, and musculoskeletal symptoms, creating a complex clinical overlap [168,169]. Fatigue, a hallmark symptom in both scenarios, may be attributed to the prolonged immune response and inflammatory milieu induced by CMV reactivation and SARS-CoV-2 infection. Cognitive impairments, commonly referred to as “brain fog,” are also reported in both Long COVID and CMV reactivation, possibly linked to the neuroinflammatory effects of these viral infections. Additionally, musculoskeletal symptoms, such as joint pain and muscle aches, are frequently observed in both conditions, highlighting the systemic impact of viral-induced inflammation. Recognizing these parallels provides valuable insights into potential shared mechanisms and reinforces the need for comprehensive investigations into the long-term effects of viral infections on human health. Distinguishing between these conditions may require specific diagnostic tests and clinical evaluation.

Therefore, it is important to note that our understanding of Long COVID is still evolving, and while CMV may be a relevant factor in some cases, it is unlikely to be the sole contributor. Long COVID is a complex condition with a range of symptoms that can vary widely among individuals. Additional research is needed to determine the extent to which CMV and other factors play a role in the development and persistence of Long COVID. Clinicians and researchers continue to work together to better understand and address this condition.

## 9. Conclusions, Limitations, and Future Directions

The intricate interplay between immunosenescence and CMV underscores the profound impact of chronic viral infections on aging, health, and disease. This review has briefly outlined the multifaceted consequences of CMV infection, elucidating how it can exacerbate immunosenescence and the potential implications for age-related diseases. Specifically, the expansion of late differentiated T cells and alterations in immune cell functions, combined with the proinflammatory milieu fostered by CMV, could underlie the increased susceptibility to infections, reduced vaccine efficacy, and potentially even the development of certain cancers. Moreover, the complex relationship between CMV, immunosenescence, and SARS-CoV-2 infection underlines the relevance of understanding these interactions in the context of contemporary health challenges, such as the COVID-19 and post-COVID conditions. While significant progress has been made in understanding the interplay between immunosenescence and CMV, several limitations exist in current research, pointing to avenues for future investigation:

*Heterogeneity in study populations:* Many studies focus on specific age groups or populations, leading to a lack of comprehensive understanding of the diverse effects of CMV on immunosenescence across different demographics. Future research should encompass broader age ranges and diverse cohorts to capture the nuances of this complex relationship.

*Longitudinal studies:* The majority of existing research relies on cross-sectional data, providing snapshots of immunosenescence and CMV interactions at specific time points. Longitudinal studies are crucial for tracking the dynamic changes over time, identifying key transitional phases, and elucidating the causal relationships between CMV infection and immunosenescence.

*Genetic variability:* The influence of individual genetic variations on the response to CMV and the trajectory of immunosenescence remains understudied. Investigating the genetic determinants that shape susceptibility to CMV infection and impact immune responses will enhance our understanding of personalized risk profiles.

*In-depth epigenetic analysis:* Epigenetic modifications play a pivotal role in regulating gene expression during CMV latency and immunosenescence. Future studies should employ advanced techniques to decipher how epigenetic alterations contribute to the bidirectional relationship and influence overall immune function.

*Host–virus interactions at the single-cell level:* Advancements in single-cell technologies allow for a more specific examination of host–virus interactions. Exploring these interactions at the single-cell level can unravel heterogeneity within cell populations, providing insights into how specific cells contribute to immunosenescence and respond to CMV.

*Impact on vaccination:* Understanding the impact of CMV on vaccine responses, especially in older adults, is critical. Investigating how immunosenescence, combined with CMV infection, influences vaccine efficacy and the development of adaptive immunity is essential for optimizing vaccination strategies for the aging population.

*Impact on age-related diseases:* Further research is needed to comprehensively elucidate the impact of CMV on age-related diseases and to explore potential interventions that may mitigate the adverse effects of CMV infection. A deeper understanding of these processes is essential for enhancing the health and quality of life of the aging population and for developing more effective strategies to combat age-related diseases and infections.

*Neurological consequences:* The potential impact of CMV on neurodegenerative disorders and cognitive decline requires further attention. Longitudinal studies exploring the association between CMV infection, immunosenescence, and neurological outcomes will contribute to our understanding of age-related cognitive health.

*Intervention strategies:* While this review has shed light on the consequences of the immunosenescence–CMV interplay, research on potential interventions is limited. Future studies should explore therapeutic avenues that target both immunosenescence and CMV, aiming to enhance immune function and mitigate age-associated diseases.

*Developing CMV vaccines and anti-viral treatments:* Developing CMV vaccines that target both primary and latent infections may be a pivotal goal. Such vaccines could have a significant impact on preventing CMV-associated immunosenescence and its consequences. Investigating novel antiviral treatments for controlling CMV reactivation in aging populations is also essential. These therapies should aim to reduce the burden of CMV and its potential to drive immunosenescence. Additionally, immunomodulatory approaches may be imperative. Exploring interventions that modulate the immune response to CMV and mitigate its negative effects on immunosenescence may represent an exciting avenue for future research. These strategies may include, for instance, immune checkpoint inhibitors or immunostimulatory agents.

*Influence of co-infection:* The interactions between CMV and other pathogens, particularly in the context of co-infections, pose unresolved questions. Investigating how concurrent infections may synergistically or antagonistically influence immunosenescence is crucial for a holistic understanding of the aging immune system. As new infectious diseases continue to emerge, understanding how CMV affects immune responses to these pathogens is crucial. Insights into how CMV interacts with novel viruses, such as the ongoing research on its influence on SARS-CoV-2 infection, remains essential.

Thus, while considerable progress has been made in unraveling the mechanisms at the interface of CMV and immunosenescence, many questions remain. The direct impact of CMV on longevity and health span remains unclear. Research focusing on the relationship between CMV infection, aging, and overall health outcomes can contribute to a comprehensive understanding of its implications for the aging population. The identification of reliable biomarkers for CMV-associated immunosenescence and related diseases is pivotal. Such biomarkers would enable early detection and monitoring, facilitating timely interventions. Addressing these limitations may contribute to a more comprehensive understanding of the intricate relationship between immunosenescence and CMV. This knowledge is crucial for improving the health and well-being of aging individuals and advancing our knowledge of age-related diseases and infections.

## Data Availability

Not applicable.

## References

[1] Pawelec G. (2018). Age and immunity: What is “immunosenescence”?. Exp. Gerontol..

[2] Nikolich-Zugich J. (2018). The twilight of immunity: Emerging concepts in aging of the immune system. Nat. Immunol..

[3] Pangrazzi L., Weinberger B. (2020). T cells, aging and senescence. Exp. Gerontol..

[4] Müller L., Di Benedetto S., Pawelec G. (2019). The Immune System and Its Dysregulation with Aging. Subcell. Biochem..

[5] Müller L., Fulop T., Pawelec G. (2013). Immunosenescence in vertebrates and invertebrates. Immun. Ageing.

[6] Liu Z., Liang Q., Ren Y., Guo C., Ge X., Wang L., Cheng Q., Luo P., Zhang Y., Han X. (2023). Immunosenescence: Molecular mechanisms and diseases. Signal Transduct. Target. Ther..

[7] Mittelbrunn M., Kroemer G. (2021). Hallmarks of T cell aging. Nat. Immunol..

[8] Franceschi C., Bonafe M., Valensin S., Olivieri F., De Luca M., Ottaviani E., De Benedictis G. (2000). Inflamm-aging. An evolutionary perspective on immunosenescence. Ann. N. Y. Acad. Sci..

[9] Aiello A., Farzaneh F., Candore G., Caruso C., Davinelli S., Gambino C.M., Ligotti M.E., Zareian N., Accardi G. (2019). Immunosenescence and Its Hallmarks: How to Oppose Aging Strategically? A Review of Potential Options for Therapeutic Intervention. Front. Immunol..

[10] Müller L., Di Benedetto S. (2021). How Immunosenescence and Inflammaging May Contribute to Hyperinflammatory Syndrome in COVID-19. Int. J. Mol. Sci..

[11] Di Benedetto S., Derhovanessian E., Steinhagen-Thiessen E., Goldeck D., Muller L., Pawelec G. (2015). Impact of age, sex and CMV-infection on peripheral T cell phenotypes: Results from the Berlin BASE-II Study. Biogerontology.

[12] Di Benedetto S., Gaetjen M., Muller L. (2019). The Modulatory Effect of Gender and Cytomegalovirus-Seropositivity on Circulating Inflammatory Factors and Cognitive Performance in Elderly Individuals. Int. J. Mol. Sci..

[13] Griffiths P., Baraniak I., Reeves M. (2015). The pathogenesis of human cytomegalovirus. J. Pathol..

[14] Heath J.J., Michael D. (2020). The Immune Response Against Human Cytomegalovirus Links Cellular to Systemic Senescence. Cells.

[15] Kadambari S., Klenerman P., Pollard A.J. (2020). Why the elderly appear to be more severely affected by COVID-19: The potential role of immunosenescence and CMV. Rev. Med. Virol..

[16] La Rosa C., Diamond D.J. (2012). The immune response to human CMV. Future Virol..

[17] Mathei C., Vaes B., Wallemacq P., Degryse J. (2011). Associations between cytomegalovirus infection and functional impairment and frailty in the BELFRAIL Cohort. J. Am. Geriatr. Soc..

[18] Wikby A., Johansson B., Olsson J., Lofgren S., Nilsson B.O., Ferguson F. (2002). Expansions of peripheral blood CD8 T-lymphocyte subpopulations and an association with cytomegalovirus seropositivity in the elderly: The Swedish NONA immune study. Exp. Gerontol..

[19] Chen Y., Klein S.L., Garibaldi B.T., Li H., Wu C., Osevala N.M., Li T., Margolick J.B., Pawelec G., Leng S.X. (2021). Aging in COVID-19: Vulnerability, immunity and intervention. Ageing Res. Rev..

[20] Golubev A.G. (2020). COVID-19: A Challenge to Physiology of Aging. Front. Physiol..

[21] Koff W.C., Williams M.A. (2020). Covid-19 and Immunity in Aging Populations—A New Research Agenda. N. Engl. J. Med..

[22] Jo N., Zhang R., Ueno H., Yamamoto T., Weiskopf D., Nagao M., Yamanaka S., Hamazaki Y. (2021). Aging and CMV Infection Affect Pre-existing SARS-CoV-2-Reactive CD8+ T Cells in Unexposed Individuals. Front. Aging.

[23] Moss P. (2020). “The ancient and the new”: Is there an interaction between cytomegalovirus and SARS-CoV-2 infection?. Immun. Ageing.

[24] Ong D.S.Y., Spitoni C., Klein Klouwenberg P.M.C., Verduyn Lunel F.M., Frencken J.F., Schultz M.J., van der Poll T., Kesecioglu J., Bonten M.J.M., Cremer O.L. (2016). Cytomegalovirus reactivation and mortality in patients with acute respiratory distress syndrome. Intensive Care Med..

[25] Fulop T., Larbi A., Pawelec G., Khalil A., Cohen A.A., Hirokawa K., Witkowski J.M., Franceschi C. (2021). Immunology of Aging: The Birth of Inflammaging. Clin. Rev. Allergy Immunol..

[26] Gruver A.L., Hudson L.L., Sempowski G.D. (2007). Immunosenescence of ageing. J. Pathol..

[27] Weiskopf D., Weinberger B., Grubeck-Loebenstein B. (2009). The aging of the immune system. Transpl. Int..

[28] Dodig S., Cepelak I., Pavic I. (2019). Hallmarks of senescence and aging. Biochem. Med..

[29] Zhang H., Weyand C.M., Goronzy J.J. (2021). Hallmarks of the aging T-cell system. FEBS J..

[30] Weksler M.E. (2000). Changes in the B-cell repertoire with age. Vaccine.

[31] Wang J., Geiger H., Rudolph K.L. (2011). Immunoaging induced by hematopoietic stem cell aging. Curr. Opin. Immunol..

[32] Aspinall R., Andrew D. (2000). Thymic involution in aging. J. Clin. Immunol..

[33] Mitchell W.A., Lang P.O., Aspinall R. (2010). Tracing thymic output in older individuals. Clin. Exp. Immunol..

[34] Weng N.P. (2006). Aging of the immune system: How much can the adaptive immune system adapt?. Immunity.

[35] Cancro M.P. (2020). Age-Associated B Cells. Annu. Rev. Immunol..

[36] Frasca D., Diaz A., Romero M., Blomberg B.B. (2016). The generation of memory B cells is maintained, but the antibody response is not, in the elderly after repeated influenza immunizations. Vaccine.

[37] Boucher N., Dufeu-Duchesne T., Vicaut E., Farge D., Effros R.B., Schachter F. (1998). CD28 expression in T cell aging and human longevity. Exp. Gerontol..

[38] Vallejo A.N. (2005). CD28 extinction in human T cells: Altered functions and the program of T-cell senescence. Immunol. Rev..

[39] Blank C.U., Haining W.N., Held W., Hogan P.G., Kallies A., Lugli E., Lynn R.C., Philip M., Rao A., Restifo N.P. (2019). Defining ‘T cell exhaustion’. Nat. Rev. Immunol..

[40] Wherry E.J. (2011). T cell exhaustion. Nat. Immunol..

[41] Shaw A.C., Joshi S., Greenwood H., Panda A., Lord J.M. (2010). Aging of the innate immune system. Curr. Opin. Immunol..

[42] Masselli E., Vaccarezza M., Carubbi C., Pozzi G., Presta V., Mirandola P., Vitale M. (2020). NK cells: A double edge sword against SARS-CoV-2. Adv. Biol. Regul..

[43] van Eeden C., Khan L., Osman M.S., Cohen Tervaert J.W. (2020). Natural Killer Cell Dysfunction and Its Role in COVID-19. Int. J. Mol. Sci..

[44] Seidler S., Zimmermann H.W., Bartneck M., Trautwein C., Tacke F. (2010). Age-dependent alterations of monocyte subsets and monocyte-related chemokine pathways in healthy adults. BMC Immunol..

[45] Wong C., Goldstein D.R. (2013). Impact of aging on antigen presentation cell function of dendritic cells. Curr. Opin. Immunol..

[46] Franceschi C., Garagnani P., Vitale G., Capri M., Salvioli S. (2017). Inflammaging and ‘Garb-aging’. Trends Endocrinol. Metab..

[47] Thomas R., Wang W., Su D.M. (2020). Contributions of Age-Related Thymic Involution to Immunosenescence and Inflammaging. Immun. Ageing.

[48] Soegiarto G., Purnomosari D. (2023). Challenges in the Vaccination of the Elderly and Strategies for Improvement. Pathophysiology.

[49] Pera A., Campos C., Lopez N., Hassouneh F., Alonso C., Tarazona R., Solana R. (2015). Immunosenescence: Implications for response to infection and vaccination in older people. Maturitas.

[50] Fulop T., Larbi A., Pawelec G., Cohen A.A., Provost G., Khalil A., Lacombe G., Rodrigues S., Desroches M., Hirokawa K. (2022). Immunosenescence and Altered Vaccine Efficiency in Older Subjects: A Myth Difficult to Change. Vaccines.

[51] Pereira B., Xu X.N., Akbar A.N. (2020). Targeting Inflammation and Immunosenescence to Improve Vaccine Responses in the Elderly. Front. Immunol..

[52] Cox L.S., Bellantuono I., Lord J.M., Sapey E., Mannick J.B., Partridge L., Gordon A.L., Steves C.J., Witham M.D. (2020). Tackling immunosenescence to improve COVID-19 outcomes and vaccine response in older adults. Lancet Healthy Longev..

[53] Pawelec G., Weng N.P. (2020). Can an effective SARS-CoV-2 vaccine be developed for the older population?. Immun. Ageing.

[54] Weinberger B. (2021). Vaccination of older adults: Influenza, pneumococcal disease, herpes zoster, COVID-19 and beyond. Immun. Ageing.

[55] Pawelec G., Akbar A., Caruso C., Solana R., Grubeck-Loebenstein B., Wikby A. (2005). Human immunosenescence: Is it infectious?. Immunol. Rev..

[56] Nikolich-Zugich J., Knox K.S., Rios C.T., Natt B., Bhattacharya D., Fain M.J. (2020). SARS-CoV-2 and COVID-19 in older adults: What we may expect regarding pathogenesis, immune responses, and outcomes. Geroscience.

[57] Torales J., O’Higgins M., Castaldelli-Maia J.M., Ventriglio A. (2020). The outbreak of COVID-19 coronavirus and its impact on global mental health. Int. J. Soc. Psychiatry.

[58] van Eijk L.E., Binkhorst M., Bourgonje A.R., Offringa A.K., Mulder D.J., Bos E.M., Kolundzic N., Abdulle A.E., van der Voort P.H., Olde Rikkert M.G. (2021). COVID-19: Immunopathology, pathophysiological mechanisms, and treatment options. J. Pathol..

[59] Zheng M., Gao Y., Wang G., Song G., Liu S., Sun D., Xu Y., Tian Z. (2020). Functional exhaustion of antiviral lymphocytes in COVID-19 patients. Cell. Mol. Immunol..

[60] Zheng Y., Liu X., Le W., Xie L., Li H., Wen W., Wang S., Ma S., Huang Z., Ye J. (2020). A human circulating immune cell landscape in aging and COVID-19. Protein Cell.

[61] Li Y., Wang C., Peng M. (2021). Aging Immune System and Its Correlation With Liability to Severe Lung Complications. Front. Public Health.

[62] Rowe T.A., Juthani-Mehta M. (2013). Urinary tract infection in older adults. Aging Health.

[63] Garbe J.C., Pepin F., Pelissier F.A., Sputova K., Fridriksdottir A.J., Guo D.E., Villadsen R., Park M., Petersen O.W., Borowsky A.D. (2012). Accumulation of multipotent progenitors with a basal differentiation bias during aging of human mammary epithelia. Cancer Res..

[64] Pelissier Vatter F.A., Schapiro D., Chang H., Borowsky A.D., Lee J.K., Parvin B., Stampfer M.R., LaBarge M.A., Bodenmiller B., Lorens J.B. (2018). High-Dimensional Phenotyping Identifies Age-Emergent Cells in Human Mammary Epithelia. Cell Rep..

[65] Liu Y., Sanoff H.K., Cho H., Burd C.E., Torrice C., Ibrahim J.G., Thomas N.E., Sharpless N.E. (2009). Expression of p16(INK4a) in peripheral blood T-cells is a biomarker of human aging. Aging Cell.

[66] Weyand C.M., Fulbright J.W., Goronzy J.J. (2003). Immunosenescence, autoimmunity, and rheumatoid arthritis. Exp. Gerontol..

[67] Montoya-Ortiz G. (2013). Immunosenescence, aging, and systemic lupus erythematous. Autoimmune Dis..

[68] Franceschi C., Garagnani P., Parini P., Giuliani C., Santoro A. (2018). Inflammaging: A new immune-metabolic viewpoint for age-related diseases. Nat. Rev. Endocrinol..

[69] Perdaens O., van Pesch V. (2021). Molecular Mechanisms of Immunosenescene and Inflammaging: Relevance to the Immunopathogenesis and Treatment of Multiple Sclerosis. Front. Neurol..

[70] Zhao T.V., Sato Y., Goronzy J.J., Weyand C.M. (2022). T-Cell Aging-Associated Phenotypes in Autoimmune Disease. Front. Aging.

[71] Müller L., Di Benedetto S. (2023). From aging to long COVID: Exploring the convergence of immunosenescence, inflammaging, and autoimmunity. Front. Immunol..

[72] Prelog M. (2006). Aging of the immune system: A risk factor for autoimmunity?. Autoimmun. Rev..

[73] Poole E., Sinclair J. (2015). Sleepless latency of human cytomegalovirus. Med. Microbiol. Immunol..

[74] Forte E., Zhang Z., Thorp E.B., Hummel M. (2020). Cytomegalovirus Latency and Reactivation: An Intricate Interplay With the Host Immune Response. Front. Cell Infect Microbiol..

[75] Tu W., Rao S. (2016). Mechanisms Underlying T Cell Immunosenescence: Aging and Cytomegalovirus Infection. Front. Microbiol..

[76] Wikby A., Ferguson F., Forsey R., Thompson J., Strindhall J., Lofgren S., Nilsson B.O., Ernerudh J., Pawelec G., Johansson B. (2005). An immune risk phenotype, cognitive impairment, and survival in very late life: Impact of allostatic load in Swedish octogenarian and nonagenarian humans. J. Gerontol. A Biol. Sci. Med. Sci..

[77] Goodrum F. (2016). Human Cytomegalovirus Latency: Approaching the Gordian Knot. Annu. Rev. Virol..

[78] Balthesen M., Dreher L., Lucin P., Reddehase M.J. (1994). The establishment of cytomegalovirus latency in organs is not linked to local virus production during primary infection. J. Gen. Virol..

[79] O’Connor C.M. (2021). Cytomegalovirus (CMV) Infection and Latency. Pathogens.

[80] Dupont L., Reeves M.B. (2016). Cytomegalovirus latency and reactivation: Recent insights into an age old problem. Rev. Med. Virol..

[81] Taylor-Wiedeman J., Sissons J.G., Borysiewicz L.K., Sinclair J.H. (1991). Monocytes are a major site of persistence of human cytomegalovirus in peripheral blood mononuclear cells. J. Gen. Virol..

[82] Wills M.R., Poole E., Lau B., Krishna B., Sinclair J.H. (2015). The immunology of human cytomegalovirus latency: Could latent infection be cleared by novel immunotherapeutic strategies?. Cell. Mol. Immunol..

[83] Seckert C.K., Renzaho A., Tervo H.M., Krause C., Deegen P., Kuhnapfel B., Reddehase M.J., Grzimek N.K. (2009). Liver sinusoidal endothelial cells are a site of murine cytomegalovirus latency and reactivation. J. Virol..

[84] Balthesen M., Messerle M., Reddehase M.J. (1993). Lungs are a major organ site of cytomegalovirus latency and recurrence. J. Virol..

[85] Nikolich-Zugich J., Cicin-Sain L., Collins-McMillen D., Jackson S., Oxenius A., Sinclair J., Snyder C., Wills M., Lemmermann N. (2020). Advances in cytomegalovirus (CMV) biology and its relationship to health, diseases, and aging. Geroscience.

[86] Pera A., Campos C., Corona A., Sanchez-Correa B., Tarazona R., Larbi A., Solana R. (2014). CMV latent infection improves CD8+ T response to SEB due to expansion of polyfunctional CD57+ cells in young individuals. PLoS ONE.

[87] Precopio M.L., Betts M.R., Parrino J., Price D.A., Gostick E., Ambrozak D.R., Asher T.E., Douek D.C., Harari A., Pantaleo G. (2007). Immunization with vaccinia virus induces polyfunctional and phenotypically distinctive CD8(+) T cell responses. J. Exp. Med..

[88] Santos Rocha C., Hirao L.A., Weber M.G., Mendez-Lagares G., Chang W.L.W., Jiang G., Deere J.D., Sparger E.E., Roberts J., Barry P.A. (2018). Subclinical Cytomegalovirus Infection Is Associated with Altered Host Immunity, Gut Microbiota, and Vaccine Responses. J. Virol..

[89] Vallejo A.N., Brandes J.C., Weyand C.M., Goronzy J.R.J. (1999). Modulation of CD28 Expression: Distinct Regulatory Pathways During Activation and Replicative Senescence1. J. Immunol..

[90] Alvarez-Heredia P., Reina-Alfonso I., Dominguez-Del-Castillo J.J., Gutierrez-Gonzalez C., Hassouneh F., Batista-Duharte A., Perez A.B., Tarazona R., Solana R., Pera A. (2023). Accelerated T-Cell Immunosenescence in Cytomegalovirus-Seropositive Individuals After Severe Acute Respiratory Syndrome Coronavirus 2 Infection. J. Infect. Dis..

[91] Sylwester A.W., Mitchell B.L., Edgar J.B., Taormina C., Pelte C., Ruchti F., Sleath P.R., Grabstein K.H., Hosken N.A., Kern F. (2005). Broadly targeted human cytomegalovirus-specific CD4+ and CD8+ T cells dominate the memory compartments of exposed subjects. J. Exp. Med..

[92] Hadrup S.R., Strindhall J., Kollgaard T., Seremet T., Johansson B., Pawelec G., thor Straten P., Wikby A. (2006). Longitudinal studies of clonally expanded CD8 T cells reveal a repertoire shrinkage predicting mortality and an increased number of dysfunctional cytomegalovirus-specific T cells in the very elderly. J. Immunol..

[93] Rotival M., Quintana-Murci L. (2023). Environmental variation and genetic diversity contribute to population differences in immune responses to SARS-CoV-2 and COVID-19 risk. Genes Immun..

[94] Liston A., Carr E.J., Linterman M.A. (2016). Shaping Variation in the Human Immune System. Trends Immunol..

[95] Liston A., Humblet-Baron S., Duffy D., Goris A. (2021). Human immune diversity: From evolution to modernity. Nat. Immunol..

[96] Goldeck D., Larsen L.A., Christensen K., Hamprecht K., Ottinger L., Hahnel K., Pawelec G. (2021). Impact of Cytomegalovirus Infection and Genetic Background on the Frequencies of Peripheral Blood Suppressor Cells in Human Twins. Pathogens.

[97] Sharifi-Rad M., Anil Kumar N.V., Zucca P., Varoni E.M., Dini L., Panzarini E., Rajkovic J., Tsouh Fokou P.V., Azzini E., Peluso I. (2020). Lifestyle, Oxidative Stress, and Antioxidants: Back and Forth in the Pathophysiology of Chronic Diseases. Front. Physiol..

[98] Furman D., Campisi J., Verdin E., Carrera-Bastos P., Targ S., Franceschi C., Ferrucci L., Gilroy D.W., Fasano A., Miller G.W. (2019). Chronic inflammation in the etiology of disease across the life span. Nat. Med..

[99] Hummel M., Abecassis M.M. (2002). A model for reactivation of CMV from latency. J. Clin. Virol..

[100] Prosch S., Staak K., Stein J., Liebenthal C., Stamminger T., Volk H.D., Kruger D.H. (1995). Stimulation of the human cytomegalovirus IE enhancer/promoter in HL-60 cells by TNFalpha is mediated via induction of NF-kappaB. Virology.

[101] Haspot F., Lavault A., Sinzger C., Laib Sampaio K., Stierhof Y.D., Pilet P., Bressolette-Bodin C., Halary F. (2012). Human cytomegalovirus entry into dendritic cells occurs via a macropinocytosis-like pathway in a pH-independent and cholesterol-dependent manner. PLoS ONE.

[102] Freeman R.B. (2009). The ‘indirect’ effects of cytomegalovirus infection. Am. J. Transplant..

[103] Solana R., Tarazona R., Aiello A.E., Akbar A.N., Appay V., Beswick M., Bosch J.A., Campos C., Cantisan S., Cicin-Sain L. (2012). CMV and Immunosenescence: From basics to clinics. Immun. Ageing.

[104] Chiu Y.L., Lin C.H., Sung B.Y., Chuang Y.F., Schneck J.P., Kern F., Pawelec G., Wang G.C. (2016). Cytotoxic polyfunctionality maturation of cytomegalovirus-pp65-specific CD4+ and CD8+ T-cell responses in older adults positively correlates with response size. Sci. Rep..

[105] Aiello A.E., Chiu Y.L., Frasca D. (2017). How does cytomegalovirus factor into diseases of aging and vaccine responses, and by what mechanisms?. Geroscience.

[106] Grahame-Clarke C., Chan N.N., Andrew D., Ridgway G.L., Betteridge D.J., Emery V., Colhoun H.M., Vallance P. (2003). Human cytomegalovirus seropositivity is associated with impaired vascular function. Circulation.

[107] Simanek A.M., Dowd J.B., Pawelec G., Melzer D., Dutta A., Aiello A.E. (2011). Seropositivity to cytomegalovirus, inflammation, all-cause and cardiovascular disease-related mortality in the United States. PLoS ONE.

[108] Tracy R.P., Doyle M.F., Olson N.C., Huber S.A., Jenny N.S., Sallam R., Psaty B.M., Kronmal R.A. (2013). T-helper type 1 bias in healthy people is associated with cytomegalovirus serology and atherosclerosis: The Multi-Ethnic Study of Atherosclerosis. J. Am. Heart Assoc..

[109] Childs B.G., Baker D.J., Wijshake T., Conover C.A., Campisi J., van Deursen J.M. (2016). Senescent intimal foam cells are deleterious at all stages of atherosclerosis. Science.

[110] Djaoud Z., Riou R., Gavlovsky P.J., Mehlal S., Bressollette C., Gerard N., Gagne K., Charreau B., Retiere C. (2016). Cytomegalovirus-Infected Primary Endothelial Cells Trigger NKG2C+ Natural Killer Cells. J. Innate. Immun..

[111] Broadley I., Pera A., Morrow G., Davies K.A., Kern F. (2017). Expansions of Cytotoxic CD4(+)CD28(−) T Cells Drive Excess Cardiovascular Mortality in Rheumatoid Arthritis and Other Chronic Inflammatory Conditions and Are Triggered by CMV Infection. Front. Immunol..

[112] Guzik T.J., Hoch N.E., Brown K.A., McCann L.A., Rahman A., Dikalov S., Goronzy J., Weyand C., Harrison D.G. (2007). Role of the T cell in the genesis of angiotensin II induced hypertension and vascular dysfunction. J. Exp. Med..

[113] Pachnio A., Ciaurriz M., Begum J., Lal N., Zuo J., Beggs A., Moss P. (2016). Cytomegalovirus Infection Leads to Development of High Frequencies of Cytotoxic Virus-Specific CD4+ T Cells Targeted to Vascular Endothelium. PLoS Pathog..

[114] Weis M., Kledal T.N., Lin K.Y., Panchal S.N., Gao S.Z., Valantine H.A., Mocarski E.S., Cooke J.P. (2004). Cytomegalovirus infection impairs the nitric oxide synthase pathway: Role of asymmetric dimethylarginine in transplant arteriosclerosis. Circulation.

[115] Firth C., Harrison R., Ritchie S., Wardlaw J., Ferro C.J., Starr J.M., Deary I.J., Moss P. (2016). Cytomegalovirus infection is associated with an increase in systolic blood pressure in older individuals. QJM.

[116] Cheng J., Ke Q., Jin Z., Wang H., Kocher O., Morgan J.P., Zhang J., Crumpacker C.S. (2009). Cytomegalovirus infection causes an increase of arterial blood pressure. PLoS Pathog..

[117] Diaz N., Minton S., Cox C., Bowman T., Gritsko T., Garcia R., Eweis I., Wloch M., Livingston S., Seijo E. (2006). Activation of stat3 in primary tumors from high-risk breast cancer patients is associated with elevated levels of activated SRC and survivin expression. Clin. Cancer Res..

[118] Herbein G., Kumar A. (2014). The oncogenic potential of human cytomegalovirus and breast cancer. Front. Oncol..

[119] Bai B., Wang X., Chen E., Zhu H. (2016). Human cytomegalovirus infection and colorectal cancer risk: A meta-analysis. Oncotarget.

[120] McFaline-Figueroa J.R., Wen P.Y. (2017). The Viral Connection to Glioblastoma. Curr. Infect. Dis. Rep..

[121] Samanta M., Harkins L., Klemm K., Britt W.J., Cobbs C.S. (2003). High prevalence of human cytomegalovirus in prostatic intraepithelial neoplasia and prostatic carcinoma. J. Urol..

[122] Joshi D., Quadri M., Gangane N., Joshi R., Gangane N. (2009). Association of Epstein Barr virus infection (EBV) with breast cancer in rural Indian women. PLoS ONE.

[123] Herbein G. (2018). The Human Cytomegalovirus, from Oncomodulation to Oncogenesis. Viruses.

[124] Cobbs C.S., Harkins L., Samanta M., Gillespie G.Y., Bharara S., King P.H., Nabors L.B., Cobbs C.G., Britt W.J. (2002). Human cytomegalovirus infection and expression in human malignant glioma. Cancer Res..

[125] El Baba R., Pasquereau S., Haidar Ahmad S., Monnien F., Abad M., Bibeau F., Herbein G. (2023). EZH2-Myc driven glioblastoma elicited by cytomegalovirus infection of human astrocytes. Oncogene.

[126] Michaelis M., Doerr H.W., Cinatl J. (2009). The story of human cytomegalovirus and cancer: Increasing evidence and open questions. Neoplasia.

[127] Harkins L., Volk A.L., Samanta M., Mikolaenko I., Britt W.J., Bland K.I., Cobbs C.S. (2002). Specific localisation of human cytomegalovirus nucleic acids and proteins in human colorectal cancer. Lancet.

[128] Yu C., He S., Zhu W., Ru P., Ge X., Govindasamy K. (2023). Human cytomegalovirus in cancer: The mechanism of HCMV-induced carcinogenesis and its therapeutic potential. Front. Cell. Infect. Microbiol..

[129] Lucas K.G., Bao L., Bruggeman R., Dunham K., Specht C. (2011). The detection of CMV pp65 and IE1 in glioblastoma multiforme. J. Neurooncol..

[130] Rahman M., Dastmalchi F., Karachi A., Mitchell D. (2019). The role of CMV in glioblastoma and implications for immunotherapeutic strategies. Oncoimmunology.

[131] Fornara O., Odeberg J., Wolmer Solberg N., Tammik C., Skarman P., Peredo I., Stragliotto G., Rahbar A., Soderberg-Naucler C. (2015). Poor survival in glioblastoma patients is associated with early signs of immunosenescence in the CD4 T-cell compartment after surgery. Oncoimmunology.

[132] Zheng H., Ford B.N., Kuplicki R., Burrows K., Hunt P.W., Bodurka J., Kent Teague T., Irwin M.R., Yolken R.H., Paulus M.P. (2021). Association between cytomegalovirus infection, reduced gray matter volume, and resting-state functional hypoconnectivity in major depressive disorder: A replication and extension. Transl. Psychiatry.

[133] Ribalta T., Martinez A.J., Jares P., Muntane J., Miquel R., Claramonte X., Cardesa A. (2002). Presence of occult cytomegalovirus infection in the brain after orthotopic liver transplantation. An autopsy study of 83 cases. Virchows Arch..

[134] Aiello A.E., Haan M., Blythe L., Moore K., Gonzalez J.M., Jagust W. (2006). The influence of latent viral infection on rate of cognitive decline over 4 years. J. Am. Geriatr. Soc..

[135] Di Benedetto S., Müller L., Rauskolb S., Sendtner M., Deutschbein T., Pawelec G., Muller V. (2019). Network topology dynamics of circulating biomarkers and cognitive performance in older Cytomegalovirus-seropositive or -seronegative men and women. Immun. Ageing.

[136] Burgdorf K.S., Trabjerg B.B., Pedersen M.G., Nissen J., Banasik K., Pedersen O.B., Sorensen E., Nielsen K.R., Larsen M.H., Erikstrup C. (2019). Large-scale study of Toxoplasma and Cytomegalovirus shows an association between infection and serious psychiatric disorders. Brain Behav. Immun..

[137] Phillips A.C., Carroll D., Khan N., Moss P. (2008). Cytomegalovirus is associated with depression and anxiety in older adults. Brain Behav. Immun..

[138] Houenou J., d’Albis M.A., Daban C., Hamdani N., Delavest M., Lepine J.P., Vederine F.E., Carde S., Lajnef M., Cabon C. (2014). Cytomegalovirus seropositivity and serointensity are associated with hippocampal volume and verbal memory in schizophrenia and bipolar disorder. Prog. Neuropsychopharmacol. Biol. Psychiatry.

[139] Carbone I., Lazzarotto T., Ianni M., Porcellini E., Forti P., Masliah E., Gabrielli L., Licastro F. (2014). Herpes virus in Alzheimer’s disease: Relation to progression of the disease. Neurobiol. Aging.

[140] Lin W.R., Wozniak M.A., Wilcock G.K., Itzhaki R.F. (2002). Cytomegalovirus is present in a very high proportion of brains from vascular dementia patients. Neurobiol. Dis..

[141] Zorrilla E.P., Luborsky L., McKay J.R., Rosenthal R., Houldin A., Tax A., McCorkle R., Seligman D.A., Schmidt K. (2001). The relationship of depression and stressors to immunological assays: A meta-analytic review. Brain Behav. Immun..

[142] Evans D.L., Ten Have T.R., Douglas S.D., Gettes D.R., Morrison M., Chiappini M.S., Brinker-Spence P., Job C., Mercer D.E., Wang Y.L. (2002). Association of depression with viral load, CD8 T lymphocytes, and natural killer cells in women with HIV infection. Am. J. Psychiatry.

[143] Glass C.K., Saijo K., Winner B., Marchetto M.C., Gage F.H. (2010). Mechanisms underlying inflammation in neurodegeneration. Cell.

[144] Gow A.J., Firth C.M., Harrison R., Starr J.M., Moss P., Deary I.J. (2013). Cytomegalovirus infection and cognitive abilities in old age. Neurobiol. Aging.

[145] Stebbins R.C., Noppert G.A., Yang Y.C., Dowd J.B., Simanek A., Aiello A.E. (2021). Association Between Immune Response to Cytomegalovirus and Cognition in the Health and Retirement Study. Am. J. Epidemiol..

[146] Torniainen-Holm M., Suvisaari J., Lindgren M., Harkanen T., Dickerson F., Yolken R.H. (2018). Association of cytomegalovirus and Epstein-Barr virus with cognitive functioning and risk of dementia in the general population: 11-year follow-up study. Brain Behav. Immun..

[147] Vivek S., Nelson H.H., Prizment A.E., Faul J., Crimmins E.M., Thyagarajan B. (2022). Cross sectional association between cytomegalovirus seropositivity, inflammation and cognitive impairment in elderly cancer survivors. Cancer Causes Control.

[148] Ludlow M., Kortekaas J., Herden C., Hoffmann B., Tappe D., Trebst C., Griffin D.E., Brindle H.E., Solomon T., Brown A.S. (2016). Neurotropic virus infections as the cause of immediate and delayed neuropathology. Acta Neuropathol..

[149] Deleidi M., Isacson O. (2012). Viral and inflammatory triggers of neurodegenerative diseases. Sci. Transl. Med..

[150] Zheng H., Ford B.N., Bergamino M., Kuplicki R., Tulsa I., Hunt P.W., Bodurka J., Teague T.K., Irwin M.R., Yolken R.H. (2021). A hidden menace? Cytomegalovirus infection is associated with reduced cortical gray matter volume in major depressive disorder. Mol. Psychiatry.

[151] Zheng H., Bergamino M., Ford B.N., Kuplicki R., Yeh F.C., Bodurka J., Burrows K., Tulsa I., Hunt P.W., Teague T.K. (2021). Replicable association between human cytomegalovirus infection and reduced white matter fractional anisotropy in major depressive disorder. Neuropsychopharmacology.

[152] Ford B.N., Savitz J. (2022). Depression, aging, and immunity: Implications for COVID-19 vaccine immunogenicity. Immun. Ageing.

[153] Di Benedetto S., Müller L., Wenger E., Duzel S., Pawelec G. (2017). Contribution of neuroinflammation and immunity to brain aging and the mitigating effects of physical and cognitive interventions. Neurosci. Biobehav. Rev..

[154] Halenius A., Hengel H. (2014). Human cytomegalovirus and autoimmune disease. BioMed Res. Int..

[155] Alanio C., Verma A., Mathew D., Gouma S., Liang G., Dunn T., Oldridge D.A., Weaver J., Kuri-Cervantes L., Pampena M.B. (2022). Cytomegalovirus Latent Infection is Associated with an Increased Risk of COVID-19-Related Hospitalization. J. Infect. Dis..

[156] Frozza F.T.B., Fazolo T., de Souza P.O., Lima K., da Fontoura J.C., Borba T.S., Polese-Bonatto M., Kern L.B., Stein R.T., Pawelec G. (2023). A high CMV-specific T cell response associates with SARS-CoV-2-specific IL-17 T cell production. Med. Microbiol. Immunol..

[157] Vescovini R., Biasini C., Telera A.R., Basaglia M., Stella A., Magalini F., Bucci L., Monti D., Lazzarotto T., Dal Monte P. (2010). Intense antiextracellular adaptive immune response to human cytomegalovirus in very old subjects with impaired health and cognitive and functional status. J. Immunol..

[158] Furman D., Jojic V., Sharma S., Shen-Orr S.S., Angel C.J., Onengut-Gumuscu S., Kidd B.A., Maecker H.T., Concannon P., Dekker C.L. (2015). Cytomegalovirus infection enhances the immune response to influenza. Sci. Transl. Med..

[159] Müller L., Di Benedetto S. (2023). Aged brain and neuroimmune responses to COVID-19: Post-acute sequelae and modulatory effects of behavioral and nutritional interventions. Immun. Ageing.

[160] Soderberg-Naucler C. (2021). Does reactivation of cytomegalovirus contribute to severe COVID-19 disease?. Immun. Ageing.

[161] Ladds E., Rushforth A., Wieringa S., Taylor S., Rayner C., Husain L., Greenhalgh T. (2020). Persistent symptoms after Covid-19: Qualitative study of 114 “long Covid” patients and draft quality principles for services. BMC Health Serv. Res..

[162] Astin R., Banerjee A., Baker M.R., Dani M., Ford E., Hull J.H., Lim P.B., McNarry M., Morten K., O’Sullivan O. (2023). Long COVID: Mechanisms, risk factors and recovery. Exp. Physiol..

[163] Hazeldine J., Lord J.M. (2020). Immunesenescence: A Predisposing Risk Factor for the Development of COVID-19?. Front. Immunol..

[164] Bartleson J.M., Radenkovic D., Covarrubias A.J., Furman D., Winer D.A., Verdin E. (2021). SARS-CoV-2, COVID-19 and the aging immune system. Nat. Aging.

[165] Phetsouphanh C., Darley D.R., Wilson D.B., Howe A., Munier C.M.L., Patel S.K., Juno J.A., Burrell L.M., Kent S.J., Dore G.J. (2022). Immunological dysfunction persists for 8 months following initial mild-to-moderate SARS-CoV-2 infection. Nat. Immunol..

[166] Ryan F.J., Hope C.M., Masavuli M.G., Lynn M.A., Mekonnen Z.A., Yeow A.E.L., Garcia-Valtanen P., Al-Delfi Z., Gummow J., Ferguson C. (2022). Long-term perturbation of the peripheral immune system months after SARS-CoV-2 infection. BMC Med..

[167] Peluso M.J., Deeks S.G. (2022). Early clues regarding the pathogenesis of long-COVID. Trends Immunol..

[168] Chaves-Filho A.M., Braniff O., Angelova A., Deng Y., Tremblay M.E. (2023). Chronic inflammation, neuroglial dysfunction, and plasmalogen deficiency as a new pathobiological hypothesis addressing the overlap between post-COVID-19 symptoms and myalgic encephalomyelitis/chronic fatigue syndrome. Brain Res. Bull..

[169] Shikova E., Reshkova V., Kumanova A.C., Raleva S., Alexandrova D., Capo N., Murovska M., On Behalf Of The European Network On Me/Cfs Euromene (2020). Cytomegalovirus, Epstein-Barr virus, and human herpesvirus-6 infections in patients with myalgic small ie, Cyrillicncephalomyelitis/chronic fatigue syndrome. J. Med. Virol..

